# Occurrence of *vanHAX* and Related Genes beyond the *Actinobacteria* Phylum

**DOI:** 10.3390/genes13111960

**Published:** 2022-10-27

**Authors:** Oleksandr Yushchuk, Elisa Binda, Victor Fedorenko, Flavia Marinelli

**Affiliations:** 1 Department of Biotechnology and Life Sciences, University of Insubria, 21100 Varese, Italy; 2 Department of Genetics and Biotechnology, Ivan Franko National University of Lviv, 79005 Lviv, Ukraine

**Keywords:** antibiotic resistance, glycopeptide resistance, *van* genes, mobile genetic elements

## Abstract

Clinically relevant glycopeptide antibiotics remain among the most successful classes of natural antibacterials. This success, however, is endangered by the spread of glycopeptide resistance genes, also known as *van* genes. Thus, it is important to trace and comprehend possible routes of *van* gene dissemination. In the current work, we present a comprehensive bioinformatic analysis aimed at mapping the occurrence of *van* genes beyond the *Actinobacteria* phylum—the most likely natural reservoir of *van* genes. We show that two additional classes of Gram-positive bacteria, *Erysipelotrichia* and *Ktedonobacteria*, as well as one class of Gram-negative bacteria, *Anaerolineae*, carry *van* genes. Additionally, we demonstrate that various new genera belonging to the classes *Clostridia* and *Bacilli* also carry *van* genes. The majority of discovered *van* loci are co-localized with MGE-related genes of various types. Finally, we propose a phylogeny-based scenario for the spread of *van* genes, unraveling a network of consequential horizontal gene transfer events linking the phylum *Actinobacteria* with the five other bacterial classes carrying *van* genes.

## 1. Introduction

Glycopeptide antibiotics—more precisely, dalbaheptides—represent a large group of natural products produced by soil-dwelling, high-G-C-content, and Gram-positive actinomycetes [1,2]. Dalbaheptides are products of sophisticated biosynthetic machinery involving the non-ribosomal synthesis of the heptapeptide aglycone, followed by numerous and variable modifications. Genes encoding these pathways are co-localized, co-expressed, and organized in large biosynthetic gene clusters (BGCs). Dalbaheptide biosynthesis has been extensively studied and reviewed in recent years [3,4,5,6,7,8,9].

Although biosynthetic and regulatory mechanisms behind the production of dalbaheptides are interesting *per se* and represent a valuable model for studying other natural products, the real importance of dalbaheptides comes from their clinical relevance [10,11,12,13]. Today, two natural (vancomycin and teicoplanin) and three semi-synthetic (dalbavancin, telavancin, and oritavancin) dalbaheptides are used to treat infections caused by multidrug-resistant (MDR) pathogens [8]. As potent inhibitors of Gram-positive cell-wall biosynthesis, dalbaheptides strongly and selectively bind lipid II [14], essential for the biosynthesis of the bacterial cell wall [15]. They specifically interact with the d-alanyl-d-alanine (d-Ala-d-Ala) termini of the lipid II pentapeptide side stems by forming five defined hydrogen bonds, thus impeding the following transpeptidation and transglycosylation steps [9,16,17].

As Gram-positive bacteria, actinobacterial dalbaheptide producers require the expression of self-resistance mechanisms to avoid suicide during antibiotic biosynthesis and excretion. Resistance mechanisms in model dalbaheptide producers have been investigated in detail in recent years [18,19,20,21]. The most relevant dalbaheptide resistance mechanism involves at least five genes—known as *van* genes (i.e., *van*comycin resistance genes)—commonly organized in two operons [22,23]. The first operon consists of three genes—*vanHAX*—coding for structural enzymes required for cell-wall remodeling. Here, VanH is an α-ketoacid dehydrogenase, transforming pyruvate to d-lactate (d-Lac); VanA is a d-Ala-d-Lac ligase, while VanX is a d,d-dipeptidase [24]. The second operon—*vanRS*—encodes a two-component regulatory system tuning *vanHAX* expression in response to the extracellular presence of a glycopeptide antibiotic. Dalbaheptide-producing actinobacteria carry *vanHAXRS* genes adjacent to or within the corresponding glycopeptide BGCs.

Based on extensive genomic analysis, we recently reported [25] that *vanHAXRS* genes are also frequently present in dalbaheptide non-producing actinobacteria, indicating that there is no unique correlation between the presence of glycopeptide BGCs and *van* genes. However, it remains unclear why dalbaheptide non-producing actinobacteria carry *vanHAXRS* genes so frequently. Actinobacteria—either dalbaheptide-producing or not—are the most likely primary environmental reservoir of *van* genes, further spreading to other bacterial taxa [25,26]. The spread of *van* genes among opportunistic pathogens such as enterococci or staphylococci represents a hot topic of investigation due to their clinical relevance [27,28,29,30,31,32,33,34,35,36,37,38,39,40,41,42,43,44,45,46,47]. On the other hand, the migration of *vanHAXRS* genes to non-harmful soil-dwelling bacteria other than *Actinobacteria* has not attracted much immediate attention, although they likely serve as a bridge to distribute *van* genes from *Actinobacteria* spp. to dangerous pathogens [26]. Previous reports highlighted that *van* genes are also present in two additional classes of Gram-positive soil bacteria, namely, the class *Bacilli* and the class *Clostridia* [48,49,50,51,52]. As reported in ESM Table S1, we analyzed in detail the precedent literature and the genomic data available on the bacterial species carrying *vanHAXRS* genes other than those belonging to the *Actinobacteria* phylum. It was observed that *vanHAXRS* genes were mainly found in enterococci (*Enterococcus* (*Ecc.*) *fecalis* and *Enterococcus faecium*) and *Staphylococcus* (*Scc.*) *aureus* strains, followed by *Paenibacillus* spp. and a few other species belonging to the *Bacilli* and *Clostridia* classes (for example, in *Clostridioides* (*Cld.*) *difficile* AI0499). In many cases, genes were found to be associated with mobile genetic elements (MGEs): transposons and plasmids (ESM Table S1), which are probably involved in their interspecies migration.

Considering the current progress in bacterial genome sequencing, in this work, we screened the vast amount of current publicly available genomic records to investigate *vanHAXRS* gene distribution in species other than those belonging to the *Actinobacteria* phylum. Our findings demonstrate for the first time that *vanHAXRS* and related genes might be found in *Anaerolineae* [53], *Erysipelotrichia* [54], and *Ktedonobacteria* [55] classes. Additional information on *van* loci spread within *Bacilli* and *Clostridia* classes is also reported. Further phylogenetic analysis of Van proteins following the one published earlier on actinobacterial Van proteins [25] adds more details to the overall picture of how *vanHAXRS* genes spread among bacterial taxa.

## 2. Methods

### 2.1. van Genes Discovery

Amino acid (aa) sequences of SCO3594, SCO3595, and SCO3596 (VanH, VanA, and VanX) from *Streptomyces* (*S*.) *coelicolor* A3(2) were used as queries for BLASTP [56] against non-redundant protein sequence databases for each bacterial class listed in the NCBI Taxonomy database [57]. The BLASTP search used default algorithm parameters, but the maximum number of aligned sequences to display was set to 5000. The analysis was performed in May 2022. All obtained hits (with no regard to the corresponding e-values) were further investigated manually and mapped to the corresponding genomic records retrieved from either GenBank [58] or RefSeq [59] databases. Every bacterial taxon in which genes coding for SCO3594-95-96 homologs were found to be co-localized was further investigated. In total, we obtained 1 set of *van* genes for *Anaerolineae*, 48 for *Bacilli*, 23 for *Clostridia*, 2 for *Erysipelotrichia*, and 7 for *Ktedonobacteria*; the accession numbers for the discovered nucleic acid and aa sequences are summarized in Supplementary Excel File S1. Metagenome-assembled genomes (MAGs) were not considered in further analyses. Degraded *van* loci (lacking either *vanH*, *vanA*, or *vanX* homologs), such as those previously observed in *Desulfitobacterium* (*Dsf.*) *hafniense* strains [52] and in some *Bacilli* spp. [25], were omitted to avoid overcomplicating the general picture.

Routine analysis with all amino and nucleic acid sequences was conducted using Geneious 4.8.5 [60].

### 2.2. Mapping MGE-Related Genes

To discover and map MGE-related genes co-localized with *van* loci, aa sequences coded by ORFs from ca. 50 kbp (or less if not available) regions up- and downstream of *van* loci were analyzed with either CD-Search or Batch CD-Search [61,62]. This allowed us to identify proteins involved in DNA transfer and recombination or prophage-related proteins. Proteins that carried conserved domains typical for IS transposases were further used as queries for BLASTP (default algorithm parameters) against the ISfinder database [63], allowing us to identify those belonging to different IS families. If necessary, inverted repeat regions were identified using the EMBOSS einverted tool [64] with the default algorithm parameters, although the “maximum extent of repeats” parameter was manually adjusted for each search to cover the whole region of interest. Putative prophage regions were inspected using Phaster [65] and PhisDetector [66] web servers under default search conditions.

The accession numbers for MGE-related genes and proteins, as well as additional information are given in Supplementary Excel File S1.

### 2.3. Phylogenetic Reconstruction

Mega 11 (v.11.0.13) [67] software was used for the phylogenetic analysis. All trees were generated using the neighbor-joining method [68]. The confidence of the branching was estimated using an interior-branch test (1000 replicates) [69]. The evolutionary distances were computed using the JTT matrix-based method [70]. Ambiguous positions were removed with the pairwise deletion option. The rate variation among sites was modeled with a 5-parameter σ distribution. The phylogeny of VanH proteins was built using 269 aa sequences, including SCO2118 (putative d-Lac dehydrogenase from *S. coelicolor* A3(2)) as an outgroup (Supplementary FASTA File S1); the phylogeny of VanA was built using 265 aa sequences proteins, including SCO5560 (putative d-Ala-d-Ala ligase from *S. coelicolor* A3(2)) as an outgroup (Supplementary FASTA File S2); the phylogeny of VanHA concatenates was constructed by using 262 aa sequences and included concatenated SCO2118-SCO5560 as an outgroup (Supplementary FASTA File S3).

## 3. Results

Our screening of publicly available genomic records revealed the presence of *vanHAXRS* and related genes in four classes of Gram-positive bacteria beyond the phylum *Actinobacteria*, i.e., *Bacilli*, *Clostridia*, *Erysipelotrichia*, and *Ktedonobacteria*, as well as in one class of Gram-negative bacteria—*Anaerolineae* (Table 1)—all belonging to the *Terrabacteria* superphylum [71]. To our best knowledge, *van* genes have not been previously reported in representatives of the last three classes. Notably, we did not include *van* loci found in MAGs in this analysis. For example, at least one MAG containing *van* loci was also found in the classes *Acidobacteria*, *Fusobacteria*, *Negativicutes*, and *Thermotogae.* However, the analysis of genes up- and downstream of these *van* loci showed them to be identical or homologous to genes from different *Actinobacteria* spp. (namely, micrococci). This allowed us to reasonably suspect anomalies in the MAG assembly, presuming that the final genome reconstruction was “contaminated” with nucleic acid sequences from other bacteria.

In the following subsections, the organization and genetic background of *vanHAXRS* for each taxonomical class is further investigated.

### 3.1. van Genes in Class Anaerolineae

**Figure 1:**
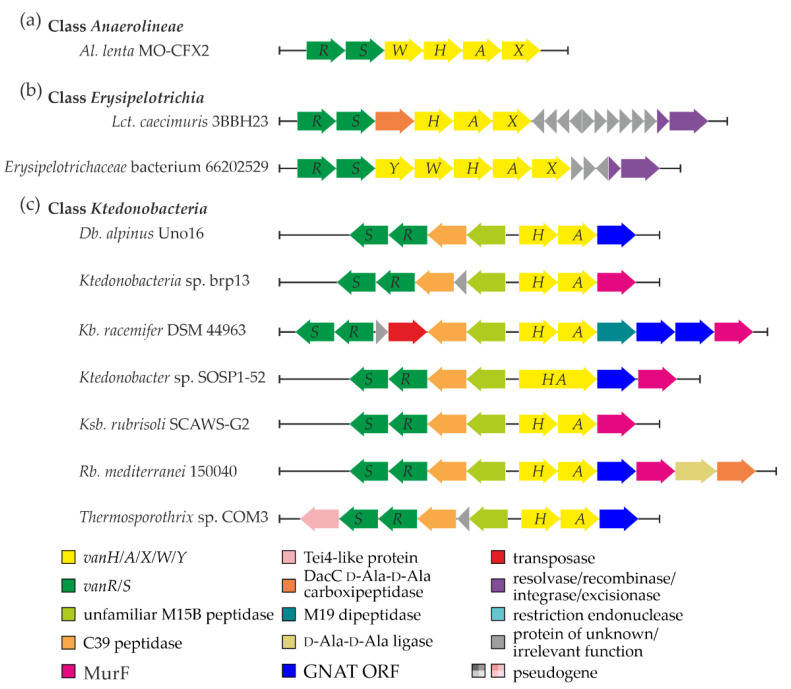
Graphical summary of *van* and other relevant co-localized genes discovered in *Anaerolineae* (**a**), *Erysipelotrichia* (**b**), and *Ktedonobacteria* (**c**) classes. The color coding of the schemes is explained in the legend of the figure. Please see the main text for more details.

A single set of *van* genes was discovered in one of the species from a still poorly investigated class of Gram-negative bacteria—*Anaerolineae* [53]. Here, the organization of *van* genes in *Al. lenta* MO-CFX2 SCF03 (*vanRSWHAX*, Figure 1a, Supplementary Excel File S1 “Anaerolineae” worksheet) resembled the one described in the *Ecc. faecalis* BM4382 pIP834^+^ plasmid-borne Tn*1549* transposon, although it lacked *vanY* homolog. In addition, a set of genes coding for putative transposases, recombinases, and integrases was found near the *van* locus (see Supplementary Excel File S1 “Anaerolineae MGERG”). However, none of them showed significant similarity to the counterparts from previously published MGEs carrying *van* genes (summarized in ESM Table S1).

### 3.2. van Genes in Class Erysipelotrichia

Most studied representatives of the class *Erysipelotrichia* belong to the gut microbiota, while the class itself was recently delineated as a sister taxon of the *Clostridia* class within the phylum *Firmicutes* [72]. Our screen indicated the presence of *van* genes in the genomes of *Lct. caecimuris* 3BBH23 (isolated from healthy human feces [73]) and of unclassified clinical *Erysipelotrichaceae* isolate 66202529 (CP046174, Supplementary Excel File S1 “Erysipelotrichia”). Both *vanRS* and *vanHAX* operons were found in *Lct. caecimuris* 3BBH23, with a gene coding for a VanYD DacC-like carboxypeptidase [74] in between (Figure 1b). The chromosome of *Erysipelotrichaceae* isolate 66202529 carried a set of *vanRSYWHAX* genes (Figure 1b), like in the *Ecc. faecium* MLG229 Tn1549-like transposon. In both *Lct. caecimuris* 3BBH23 and isolate 66202529, we found a pair of genes coding for excisionase and a tyrosine-based site-specific integrase downstream of *van* loci (Figure 1b), as well as multiple other MGE-related genes (Supplementary Excel File S1 “Erysipelotrichia”). Peculiarly, the integrase coded downstream of *van* loci in *Lct. caecimuris* 3BBH23 lacked any significant similarity to homologs in previously published MGEs carrying *van* genes (summarized in ESM Table S1). Thus, *van* loci in *Lct. caecimuris* 3BBH23 are likely to be a part of a novel MGE. On the contrary, the integrase from isolate 66202529 appeared to share 100% aa sequence identity with the integrases found downstream of *van* loci in *Ecc. faecium* MLG229 and *Clostridium* sp. MLG245 Tn*1549*-like transposons, implying that isolate 66202529 carries the latter MGE.

### 3.3. Peculiarities of Organization and Genetic Context of van Genes in Class Ktedonobacteria

For the first time, our screen revealed that *van* genes are present in representatives of the class *Ktedonobacteria* (phylum *Chloroflexi*) (Table 1, Supplementary Excel File S1 “Ktedonobacteria”). These enigmatic organisms belong to an unusual group of filamentous Gram-positive soil dwellers. *Ktedonobacteria* spp. resemble filamentous sporulating actinomycetes in certain aspects of morphology and lifestyle [55,75], implying that a sophisticated machinery for cell-wall rearrangement might exist there.

The arrangements and genetic contexts of *van* genes in *Ktedonobacteria* spp. were unprecedented: in all of them, we observed a tendency to lose *vanX* (Figure 1c, Supplementary Excel File S1 “Ktedonobacteria”). However, this loss was correlated with the gain of a novel gene pair positioned between *vanRS* and *vanHA* (Figure 1c). Remarkably, these two genes were found to code for peptidases. One peptidase belonged to the MEROPS [76] M15B subfamily of metallopeptidases, while the second resembled peptidases of the MEROPS C39 family. Typical VanX proteins also belong to the M15B subfamily but are quite different from M15B metallopeptidases from the *Ktedonobacteria* spp. mentioned above. VanX from *S. coelicolor* A3(2) (taken as a reference) shares only ca. 15% aa sequence identity with any of the *Ktedonobacteria* spp. M15B metallopeptidases, and it is shorter (202 aa vs. ca. > 320 aa). Moreover, *Kb. racemifer* DSM 44963 contained an additional gene coding for a MEROPS M19 family dipeptidase just after *vanA* (thus directly replacing the canonical position of *vanX*, Figure 1c). Other relevant genes were co-localized with *van* loci in *Ktedonobacteria* spp., including genes for MurF (a UDP-*N*-acetylmuramoyl-tripeptide ligase), a GCN5-related N-acetyltransferase (GNAT), a VanYD-like DacC d-Ala-d-Ala carboxypeptidase, and a d-Ala-d-Ala ligase (Figure 1c, Supplementary Excel File S1). Another interesting feature was observed in *Ktedonobacter* sp. SOSP1-52, where *vanH* and *vanA* were fused (Figure 1c); however, it remains unknown whether the fusion protein is produced or whether what we observed is just an error of genome sequencing and annotation.

Further analysis of the close genomic neighborhood of *van* loci in *Ktedonobacteria* spp. (except *Db. alpinus* Uno16) revealed a striking number of genes coding for transposases (often incomplete or frameshifted, Supplementary Excel File S1 “Ktedonobacteria MGERG”) belonging to different insertion sequence (IS) families. Most of them had DDE catalytic residues [77]. In addition, we discovered multiple short and long inverted repeats. Genes coding for recombinase/integrase-like enzymes were also found (Supplementary Excel File S1 “Ktedonobacteria MGERG”), although they did not show repetitive patterns across different *Ktedonobacteria* spp.

**Figure 2:**
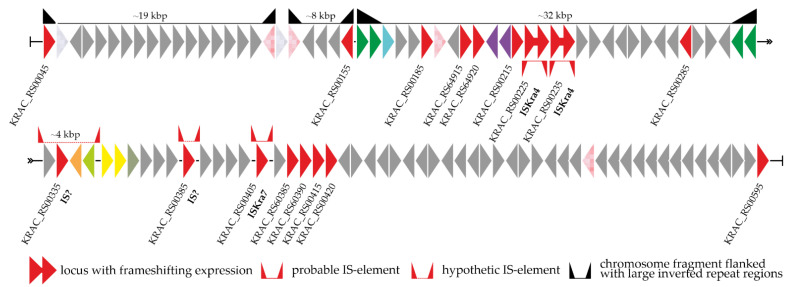
Detailed map of the genomic context of *van* loci in *Kb. racemifer* DSM 44963. The color coding of the scheme is explained in the Figure 1 legend. Please see the main text for more details.

To study the last aspect more comprehensively, we created a detailed map of the genomic neighborhood of *van* loci in *Kb. racemifer* DSM 44963 (given in Figure 2). The examined region (ca. 50 kbp up- and downstream of *van* loci) contained 21 genes for transposases (4 of them were pseudogenes), 2 genes for integrase-like enzymes, and one gene for a restriction endonuclease. Three segments surrounded by large inverted repeat regions were found upstream of the *van* loci (Figure 2): in two cases, these inverted repeats were duplicated genes for transposases, while the third case involved the duplication and inversion of the *vanRS* locus. Moreover, two intact ISKra4-like IS elements and one ISKra7-like IS element were found up- and downstream of the *van* loci, respectively. Another putative IS element composed of a gene for an IS1380-family transposase with 36 bp inverted repeats was found downstream of *van* loci; however, neither the transposase nor the repeat regions were significantly similar to any entries of the ISfinder database [63]. Finally, two peptidase genes (M15B and C39, see above) might also belong to an unknown IS element in *Kb. racemifer* DSM 44963. Both genes were co-localized with an IS200/IS605-family transposase gene and flanked with 58 bp inverted repeats (Figure 2). Interestingly, the same inverted repeats were found flanking M15B- and C39-peptidase genes in other *Ktedonobacteria* spp., although the IS200/IS605-family transposase gene was present only in *Kb. racemifer* DSM 44963.

### 3.4. Updates on vanHAXRS Gene Distribution in Bacilli Class

Bacteria belonging to the *Bacilli* class are well known to carry *vanHAXRS* genes; these are either opportunistic pathogens such as staphylococci and enterococci or non-harmful soil dwellers (see summary in ESM Table S1). Since our previous bioinformatic analysis [25], multiple novel genomic records for *Bacilli* spp. have become available in GenBank and RefSeq. This allowed us to significantly update the picture of the *vanHAXRS* distribution in the *Bacilli* class: *vanHAXRS* genes were discovered in 48 novel strains from this class (Table 1, Supplementary Excel File S1“Bacilli”).

**Figure 3:**
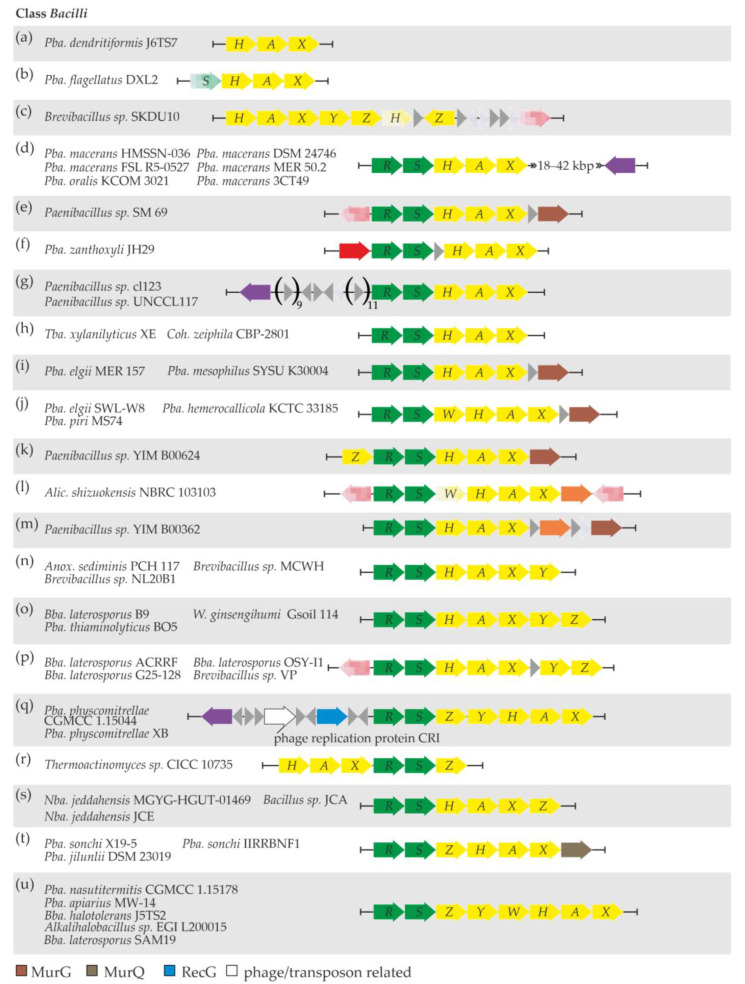
Schematic representation of the arrangement patterns observed in the *van* and relevant co-localized genes from the *Bacilli* class. Twenty one arrangement patterns (**a**–**u**) were determined and are discussed in the main text. To interpret the color coding of Figure 3, please also refer to the legend in Figure 1.

The arrangements of *van* genes varied across different strains and are summarized in Figure 3. The simplest one—only *vanHAX* genes with no MGE-related genes nearby—was discovered in *Pba. dendritiformis* J6TS7 (Figure 3a, Supplementary Excel File S1 “Bacilli”). An additional *vanS* pseudogene was present in *Pba. flagellatus* DXL2 (Figure 3b, Supplementary Excel File S1 “Bacilli”); however, *vanS* was found at the 5′-end of the available contig, suggesting that it could be intact and likely followed by a *vanR* gene. No adjacent MGE-related genes were discovered in *Pba. flagellatus* DXL2, either. The *van* genes in *Brevibacillus* sp. SKDU10 also lacked the *vanRS* locus, and they were arranged as *vanHAXYZ*. A *vanH* pseudogene, another copy of *vanZ*, and a transposase pseudogene (Figure 3c, Supplementary Excel File S1 “Bacilli”, “Bacilli MGERG”) were discovered further downstream.

A set of five *Pba. macerans* strains, together with *Pba. oralis* KCOM 3021 (Figure 3d), carried *vanRSHAX* genes with a highly conserved gene for a tyrosine-type site-specific recombinase lying downstream. This recombinase did not share any similarity with the recombinases already reported to be co-localized with *van* loci. Some other MGE-related genes were also discovered up- and downstream of *van* loci in these six strains, although they were not strictly conserved (Supplementary Excel File S1 “Bacilli MGERG”). Furthermore, *van* genes were organized as *vanRSHAX* in eight additional strains (Figure 3e–i, Supplementary Excel File S1 “Bacilli”). In addition, *van* loci were co-localized with transposase genes in *Paenibacillus* sp. SM 69 and *Pba. zanthoxyli* JH29 and with a gene for an unprecedented tyrosine-type site-specific recombinase in *Paenibacillus* sp. cl123 and *Paenibacillus* sp. UNCCL117 (Supplementary Excel File S1 “Bacilli MGERG”). Notably, in *Paenibacillus* sp. SM 69, *Pba. elgii* MER 157, and *Pba. mesophilus* SYSU K30004, a gene for UDP-*N*-acetylglucosamine-*N*-acetylmuramyl-(pentapeptide) pyrophosphoryl-undecaprenol *N*-acetylglucosamine transferase (MurG) was found downstream of *van* loci (Figure 3e,i). A slightly different arrangement—*vanRSWHAX*—was observed in three other *Paenibacillus* spp. (Figure 3j). Here, no MGE-related genes were discovered nearby, but a gene for MurG was again found downstream of *van* loci (Supplementary Excel File S1 “Bacilli”).

In *Paenibacillus* sp. YIM B00624, *van* genes were organized as *vanZRSHAX* (Figure 3k), again with no MGE-related genes nearby, but followed by a gene for MurG (Supplementary Excel File S1 “Bacilli”).

Only two strains, namely, *Alic. shizuokensis* NBRC 103103 and *Paenibacillus* sp. YIM B00362, were found to carry a gene for a DacC-like VanYD carboxypeptidase within *van* loci, organized as *vanRS(W pseudogene)HAX(YD)* and *vanRSHAX(YD)*, respectively (Figure 3l,m). In *Alic. shizuokensis* NBRC 103103, *van* loci were surrounded by two transposase pseudogenes, while *Paenibacillus* sp. YIM B00362 had no MGE-related genes near *van* loci (Supplementary Excel File S1 “Bacilli MGERG”). As in some of the other abovementioned *Paenibacillus* spp., *Paenibacillus* sp. YIM B00362 had a gene for MurG just downstream of *van* genes (Supplementary Excel File S1 “Bacilli”).

Next, in *Anox. sediminis* PCH 117, *Brevibacillus* sp. MCWH, and *Brevibacillus* sp. NL20B1 *van* genes were organized as *vanRSHAXY* (Figure 3n). No MGE-related genes were found to co-localize with *van* genes in these three strains. One additional gene—*vanZ*—was found downstream of *vanRSHAXY* in *Bba. laterosporus* B9, *Pba. thiaminolyticus* BO5, and *W. ginsengihumi Gsoil* 114 (Figure 3o). Notably, in *Bba. laterosporus* B9, *van* loci were carried on a plasmid. Another group of four *Brevibacillus* spp. had a similar arrangement of *van* genes: *vanRSHAX* and *vanYZ* loci separated by a non-correlated gene (Figure 3p), also sharing a transposase pseudogene just upstream of *vanR* (Supplementary Excel File S1 “Bacilli MGERG”).

*Pba. physcomitrellae* CGMCC 1.15044 and *Pba. physcomitrellae* XB represented a rather unique case among *Bacilli* spp., where *van* genes were organized as *vanRSZYHAX*, and a set of three MGE-related genes was found upstream (Figure 3q). These three genes code for an ATP-dependent DNA helicase RecG, a CRI phage replication protein, and an unprecedented serine-type recombinase (Supplementary Excel File S1 “Bacilli MGERG”). The RecG and recombinase proteins shared high similarity with counterparts in the genomes of phages of the order *Caudovirales* [78].

Three distinct combinations of *vanH*, *A*, *X*, *R*, *S*, and *Z* genes were observed in the following strains: *vanHAXRSZ* in *Thermoactinomyces* sp. CICC 10735 (Figure 3r); *vanRSHAXZ* in *Nba. jeddahensis* MGYG-HGUT-01469, *Nba. jeddahensis* JCE, and *Bacillus* sp. JCA (Figure 3s); and *vanRSZHAX* in *Pba. sonchi* X19-5, *Pba. sonchi* IIRRBNF1, and *Pba. jilunlii* DSM 23019 (Figure 3t). In *Thermoactinomyces* sp. CICC 10735, *Nba. jeddahensis* MGYG-HGUT-01469, *Nba. jeddahensis* JCE, *Pba. sonchi* X19-5, and *Pba. jilunlii* DSM 23019, *van* loci were not accompanied by MGE-related genes, while the genomic neighborhood of *van* genes in *Pba. sonchi* IIRRBNF1 and *Bacillus* sp. JCA was enriched with genes and pseudogenes for transposases of different types (Supplementary Excel File S1 “Bacilli MGERG”). In *Pba. sonchi* X19-5, *Pba. sonchi* IIRRBNF1, and *Pba. jilunlii* DSM 23019, a gene coding for an *N*-acetylmuramic acid 6-phosphate etherase (MurQ) was found just downstream of *vanX*.

Finally, the *vanRSZYWHAX* arrangement was discovered in *Pba. nasutitermitis* CGMCC 1.15178, *Pba. apiarius* MW-14, *Bba. halotolerans* J5TS2, *Alkalihalobacillus* sp. EGI L200015, and *Bba. laterosporus* SAM19 (Figure 3u). In each of these strains, *van* loci were accompanied by variable sets of genes and pseudogenes for transposases of different types (Supplementary Excel File S1 “Bacilli MGERG”). Notably, a gene for an unprecedented tyrosine-type site-specific integrase was found downstream of *van* loci in *Pba.* *apiarius* MW-14.

### 3.5. Updating the Picture of vanHAXRS Gene Distribution in Clostridia Class

To date, at least two *Clostridia* spp. carrying *vanHAX* and *vanRS* loci as a part of a Tn*1549*-like transposon have been reported (ESM Table S1). Bizarrely recombined *van* loci lacking *vanH* orthologs were also discovered in different strains of *Dsf. hafniense* [52] (ESM Table S1). Our screen added new results to previous data: *vanHAX*, *vanRS*, and related genes were found within the genomic records of 23 strains from the *Clostridia* class (Table 1, Supplementary Excel File S1 “Clostridia”). The arrangements of *vanHAX*, *vanRS*, and related genes were found to be quite variable.

**Figure 4:**
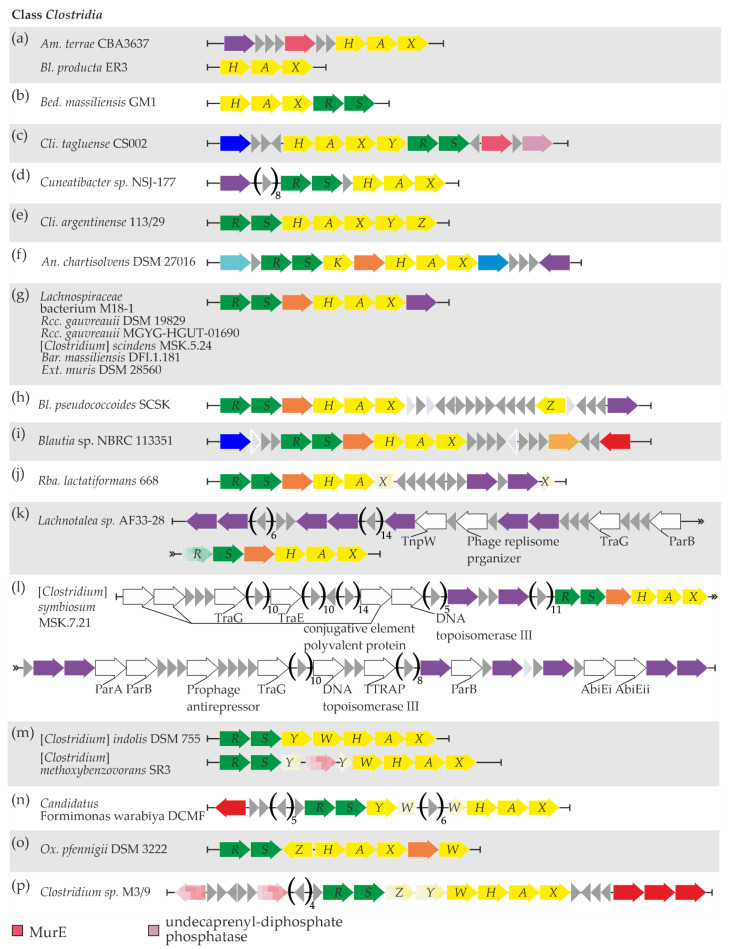
Sixteen (**a**–**p**) arrangement patterns of *van* genes and relevant neighboring genes were discovered in bacteria from the *Clostridia* class. The contents of the figure are discussed in the main text. To interpret the color coding of Figure 4, please also refer to the legends in Figure 1 and Figure 3.

The simplest one—the *vanHAX* locus only—was observed in *Am. terrae* CBA3637 and *Bl*. *producta* ER3 (Figure 4a, Supplementary Excel File S1 “Clostridia”). Additionally, in *Am. terrae* CBA3637, genes for UDP-*N*-acetylmuramoyl-l-alanyl-d-glutamate-2,6-diaminopimelate ligase (MurE) and a serine-based recombinase were found upstream of *vanHAX* (Figure 4a, Supplementary Excel File S1 “Clostridia” and “Clostridia MGERG”). This recombinase did not share any similarity with other *van*-loci-associated recombinases published previously.

The *vanHAXRS* arrangement was found in *Bed. massiliensis* GM1 (Figure 4b, Supplementary Excel File S1 “Clostridia”), although no MGE-related genes were found nearby.

Next, in *Cli. tagluense* CS002, *van* genes were arranged as *vanHAXYRS* with additional genes for MurE and undecaprenyl-diphosphate phosphatase downstream, and no MGE-related genes were found nearby (Figure 4c, Supplementary Excel File S1 “Clostridia”).

The *vanRSHAX* arrangement was observed in *Cuneatibacter* sp. NSJ-177, with an upstream gene coding for a tyrosine-based site-specific recombinase/integrase (Figure 4d, Supplementary Excel File S1 “Clostridia” and “Clostridia MGERG”). Again, this recombinase shared no similarity to the previously reported ones associated with *van* loci.

*Cli. argentinense* 113/29 exhibited the *vanRSHAXYZ* arrangement of *van* genes, with no MGE-related genes nearby (Figure 4e, Supplementary Excel File S1 “Clostridia”).

In *An*. *chartisolvens* DSM 27016, we found the *vanRS* locus, followed by *vanK*, *vanYD* (coding for a DacC-like carboxypeptidase), and *vanHAX* (Figure 4f, Supplementary Excel File S1 “Clostridia”); the *van* genes were surrounded by genes coding for a RecG-like DNA helicase and a restriction endonuclease. Finally, a gene coding for a serine-based recombinase was found downstream of *van* loci; as in the cases above, this recombinase shared no similarity with previously published *van*-loci-associated recombinases (Supplementary Excel File S1 “Clostridia MGERG”).

A *vanRS(YD)HAX* arrangement was found within the genomic records of several *Clostridia* spp. The first group of them included *Lachnospiraceae* bacterium M18-1, *Rcc.* *gauvreauii* DSM 19829, *Rcc. gauvreauii* MGYG-HGUT-01690, [*Clostridium*] *scindens* MSK.5.24, *Bar*. *massiliensis* DFI.1.181, and *Ext*. *muris* DSM 28560 (Figure 4g, Supplementary Excel File S1 “Clostridia”). All six of these bacterial strains shared a common gene for an unknown highly conserved tyrosine-based site-specific recombinase/integrase just after *vanX*. In addition, the genomic neighborhood of van loci in *Lachnospiraceae* bacterium M18-1 and *Ext*. *muris* DSM 28560 contained a rich repertoire of putative MGE-related genes (Supplementary Excel File S1 “Clostridia MGERG”). Although many of them were homologous to genes found in phages from the order *Caudovirales* [78], neither Phaster nor PhisDetector tools were able to identify prophage regions near *van* loci in M18-1 and *Ext*. *muris* DSM 28560.

The same arrangement of *van* genes was observed in *Bl. pseudococcoides* SCSK and *Blautia* sp. NBRC 113351 (Figure 4h,i). In the first strain, an additional *vanZ* gene was found downstream of the main *van* loci (Figure 4h, Supplementary Excel File S1 “Clostridia”), as well as a gene for an unprecedented serine-based recombinase (Supplementary Excel File S1 “Clostridia MGERG”). At the same time, a gene for an IS110-family transposase was downstream of *van* loci in *Blautia* sp. NBRC 113351 (Supplementary Excel File S1 “Clostridia MGERG”). A peculiar case was observed for *Rba*. *lactatiformans* 668, where the *vanRS(YD)HAX* arrangement was interrupted by the insertion of ten genes into the open reading frame (ORF) of *vanX* (Figure 4j, Supplementary Excel File S1 “Clostridia”). Two of these genes code for a MobM-like relaxase and a serine-based recombinase, respectively (Supplementary Excel File S1 “Clostridia MGERG”); neither of them was similar to the MGE-related genes found before in association with *van* loci. Finally, the *vanRS(YD)HAX* arrangement was also found in *Lachnotalea* sp. AF33-28 and [*Clostridium*] *symbiosum* MSK.7.21 (Figure 4k,l). The genetic context of *van* loci in both of these strains was quite interesting. Multiple genes coding for MGE-related proteins were found upstream of *van* loci in *Lachnotalea* sp. AF33-28 (Supplementary Excel File S1 “Clostridia MGERG”). All of these proteins lacked counterparts in previously published MGEs carrying *van* genes but appeared similar (≥56% aa sequence identity) to proteins encoded in genomes of phages from the *Caudovirales* order [78]. When prophage identification analysis was performed on *Lachnotalea* sp. AF33-28 using Phaster, an incomplete prophage region was indeed identified upstream of *van* loci. The most similar phage for this region was identified as *Faecalibacterium* phage FP_Brigit (NC_047909). A similar situation was observed in [*Clostridium*] *symbiosum* MSK.7.21 (Supplementary Excel File S1 “Clostridia MGERG”). However, neither Phaster nor PhisDetector prophage detection tools were able to discover prophages near *van* loci in [*Clostridium*] *symbiosum* MSK.7.21.

Another arrangement—*vanRSYWHAX*—was discovered in [*Clostridium*] *indolis* DSM 755, [*Clostridium*] *methoxybenzovorans* SR3 (Figure 4m, Supplementary Excel File S1 “Clostridia”), and *Candidatus* Formimonas warabiya DCMF (Figure 4n). Notably, in [*Clostridium*] *methoxybenzovorans* SR3, the *vanY* ORF was interrupted by a transposase pseudogene, while in *Candidatus* Formimonas warabiya DCMF, the *vanW* ORF contained a six-gene insertion. An IS1634-family transposase gene was also found upstream of *van* loci in the latter strain (Supplementary Excel File S1 “Clostridia MGERG”).

*Ox. pfennigii* DSM 3222 carried a *vanRSZHAX(YD)W* arrangement (Figure 4o), with no MGE-related genes nearby.

Finally, a *vanRSZYWHAX* arrangement was observed in *Clostridium* sp. M3/9, with *vanZ* and *vanY* genes containing mutations that interrupt ORFs (Figure 4p, Supplementary Excel File S1 “Clostridia”). A set of pseudo- and functional genes for transposases was found up- and downstream of *van* loci (Supplementary Excel File S1 “Clostridia MGERG”).

### 3.6. Building a Consensus Scheme for Phylogenetic Relations between Newly Discovered van Proteins and Those from Phylum Actinobacteria

Thus, our screen revealed multiple novel *van* genes from representatives of five bacterial classes. To integrate this information, we further aimed to fulfill two goals: (a) create a reliable phylogenetic tree representing the evolutionary interrelationships among Van proteins encoded by different bacterial classes; (b) see whether the genetic context of *van* genes (i.e., co-localized MGE-related genes) correlates with their phylogeny.

To achieve these goals, we first reconstructed separate phylogenies for VanH and VanA (i) encoded within the genomes of *Actinobacteria* spp. (using datasets from our previous work [25]), (ii) from nucleic acid sequences of pathogens and non-harmful soil bacteria published before (see ESM Table S1), and (iii) discovered in the current work. Separate phylogenetic reconstructions for the two proteins were coherent (see ESM Figures S1 and S2), allowing us to reasonably assume that *vanHA* (and corresponding proteins) evolved as a single unit (with insignificant exceptions). We moved onward to the creation of a representative phylogenetic tree using concatenated sequences of VanH and VanA proteins, encoded in a single locus. Unfortunately, we could not also use VanX sequences, since all of the representatives of the *Ktedonobacteria* class lack the corresponding genes (see Section 3.3). Sequences from *Dsf. hafniense* strains were also not included, since corresponding *van* loci lack *vanH* orthologs [52].

**Figure 5:**
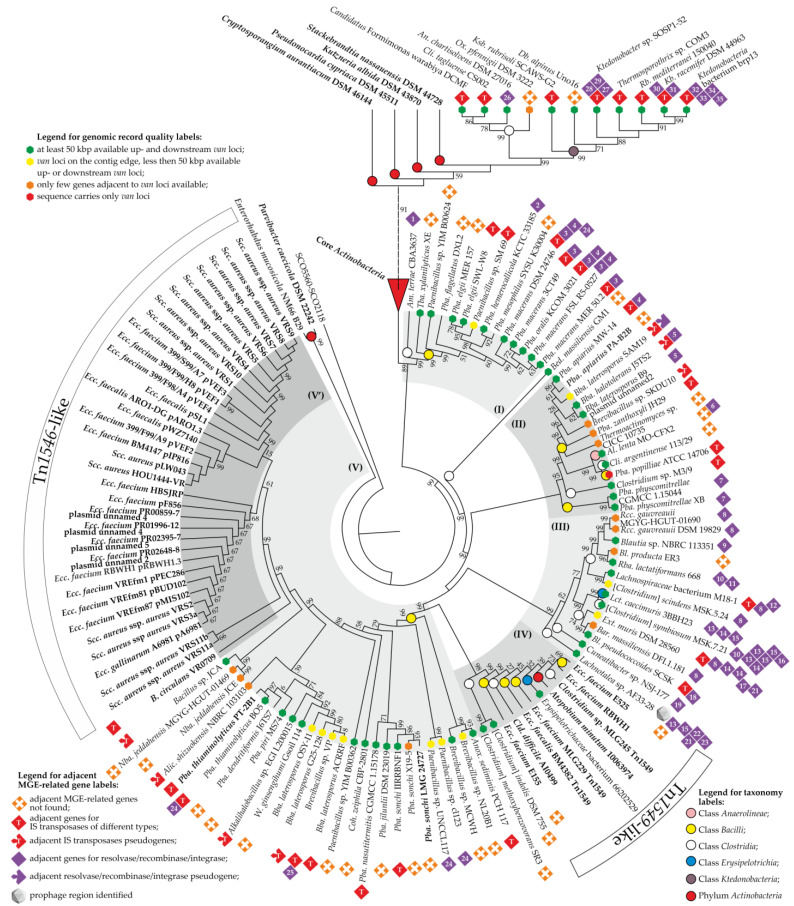
Rooted neighbor-joining phylogenetic tree of 261 VanHA sequences from *Actinobacteria*, *Anaerolineae*, *Bacilli*, *Clostridia*, *Erysipelotrichia*, and *Ktedonobacteria*, together with the sequence of SCO5560-SCO2118 used as an outgroup. The tree was generated as described in Section 2. For its better representability, branch lengths were ignored, while the “Core *Actinobacteria*” clade was collapsed; the same tree completely expanded and drawn to scale is given in ESM Figure S3. Numbers at nodes indicate bootstrap-support percentages derived from 1000 replications (shown only for ≥ 15% values). Five clades (I–V) discussed in the main text are highlighted in gray, while the labels are interpreted above and below the tree. Taxa known to carry *van* loci from previous works are given in bold. The following MGE-related enzymes are numbered 1-35 (if a numbered label is found near few taxa, the accession number would be given only for “prototype”, implying that its counterparts are close homologs sharing ≥ 50% aa sequence identity): (1) WP_162363028—serine-based recombinase/integrase; (2) WP_139603211—relaxase/mobilisation nuclease; (3) WP_124332115—tyrosine-based recombinase/integrase; (4) WP_036622915—tyrosine-based recombinase/integrase; (5) AKF95793—tyrosine-based recombinase/integrase; (6) WP_119072093—serine-based recombinase/integrase; (7) WP_124332115—serine-based recombinase/integrase; (8) WP_028530137—tyrosine-based recombinase/integrase; (9) WP_225305221—serine-based recombinase/integrase; (10) WP_016316877—relaxase/mobilisation nuclease; (11) WP_016316878—serine-based recombinase/integrase; (12) EOS38773—serine-based recombinase/integrase; (13) WP_213540055 –relaxase/mobilisation nuclease; (14) WP_213540032—tyrosine-based recombinase/integrase; (15) WP_213540058—relaxase/mobilisation nuclease; (16) WP_173892454—serine-based recombinase/integrase; (17) WP_132278971—relaxase/mobilisation nuclease; (18) WP_065543677—tyrosine-type recombinase/integrase; (19) WP_244802434—tyrosine-type recombinase/integrase; (20) WP_002569238—relaxase/mobilisation nuclease; (21) WP_117962491—serine-based recombinase/integrase; (22) WP_117761817—serine-based recombinase/integrase; (23) WP_117962497—relaxase/mobilisation nuclease; (24) WP_072327346—tyrosine-based recombinase/integrase; (25) WP_161555111—tyrosine-based recombinase/integrase; (26) WP_170138094—serine-based recombinase/integrase; (27) GHO64028—tyrosine-based recombinase/integrase; (28) GHO64041—phage integrase; (29) GHO64148—serine-based recombinase/integrase; (30) WP_007903552—tyrosine-based recombinase/integrase; (31) WP_075164122—tyrosine-based recombinase/integrase; (32) BCL79139—tyrosine-based recombinase/integrase; (33) BCL79135—serine-based recombinase/integrase; (34) BCL79150—tyrosine-based recombinase/integrase; (35) BCL79151—tyrosine-based recombinase/integrase.

The final tree is shown in Figure 5. An analysis of its topology allowed us to make some important observations. At first glance, a very recent horizontal gene transfer (HGT) event involving *van* genes from *Actinobacteria* seemed evident: VanHA sequences from all *Ktedonobacteria* spp. and from some *Clostridia* spp. (namely, *An. chartisolvens* DSM 27016, *Cli. tagluense* CS002, *Ox. pfennigii* DSM 3222, and *Candidatus* Formimonas warabiya DCMF) rooted deep in the core *Actinobacteria* clade (further referred to as “Core *Actinobacteria*”) (Figure 5), being a sister clade to VanHA from *Stackebrandtia nassauensis* DSM 44728. Notably, VanA sequences from *Dsf. hafniense* strains also belonged to an analogous clade of the VanA tree (ESM Figure S2). The repertoire of MGE-related genes co-localized with *van* loci in all of these species was variable, hindering the comprehension of transmission vectors (Figure 5, Supplementary Excel File S1 “Clostridia MGERG” and “Ktedonobacteria MGERG”). However, in *Ktedonobacteria* spp., multiple IS transposase genes were co-localized with *van* loci, as well as with seemingly intact IS elements (Supplementary Excel File S1 “Ktedonobacteria MGERG”).

In addition to the “Core *Actinobacteria*”, five major clades were defined within the tree. Clade (I) (Figure 5) appeared to be a sister clade of the “Core *Actinobacteria*”. It consisted of sequences from 12 strains of *Paenibacillus*, *Tba. xylanilyticus* XE (all 13 belonging to the class *Bacilli*), and *Am. terrae* CBA3637 (class *Clostridia*)—the last one being a basal taxon of clade (I). In all of these species, MGE-related genes co-localized with *van* loci were quite different (Figure 5, Supplementary Excel File S1 “Clostridia MGERG” and “Bacilli MGERG”). Clade (II) was composed of VanHA sequences mainly from *Bacilli* spp. These were six strains of the genus *Paenibacillus*, four strains of *Brevibacillus*, *Thermoactinomyces* sp. CICC 10735, two strains of the genus *Clostridium*, and *Al. lenta* MO-CFX2 (class *Anaerolineae*) (Figure 5). VanHA sequences from *Pba. physcomitrellae* strains formed the basal branch of clade (II). Again, here, we did not observe any reproducible pattern of MGE-related genes co-localized with *van* loci (Figure 5, Supplementary Excel File S1). Notably, it appeared that one of the clade (II) species, namely, *Bba. laterosporus* B9, carries *van* loci on a plasmid. Unfortunately, the quality of genome assemblies of another clade (II) species did not allow us to understand whether they carry *van* loci on plasmids as well.

VanHA sequences from *Clostridia* spp. (with the single exception of *Lct. caecimuris* 3BBH23, class *Erysipelotrichia*) formed clade (III) (Figure 5). The basal taxon of clade (III) was *Lachnotalea* sp. AF33-28. In contrast to clade (II), we observed a certain similarity in the repertoire of MGE-related genes co-localized with *van* loci in strains forming clade (III) (Figure 5). In some of the strains, most notably in *Lachnotalea* sp. AF33-28, [*Clostridium*] *symbiosum* MSK.7.21, *Lachnospiraceae* bacterium M18-1, and *Ext. muris* DSM 28560, large assemblages of putative MGE-related genes were associated with *van* loci (see Section 3.5). These assemblages resembled prophages, although an incomplete prophage region was identified only in *Lachnotalea* sp. AF33-28.

The last two clades—(IV) and (V)—deserve special attention. Clade (IV) was composed of VanHA sequences from [*Clostridium*] *methoxybenzovorans* SR3, [*Clostridium*] *indolis* DSM 755, *Erysipelotrichaceae* bacterium 66202529 (class *Erysipelotrichia*), and a set of strains known to carry *van* loci as a part of Tn*1549*-like transposons, namely, *Atopobium* (*Atpb*.) *minutum* 10063974 (phylum *Actinobacteria*), *Clostridium* sp. MLG245, *Cld. difficile* AI0499, *Ecc. faecium* E525, *Ecc. faecium* RBWH1, *Ecc. faecium* MLG229, *Ecc. faecalis* BM4382, and *Ecc. faecium* E155 (see ESM Table S1 for references) (Figure 5). While *Erysipelotrichaceae* bacterium 66202529 clearly carried genes from the Tn*1549*-like transposon near *van* loci (see Section 3.2), no MGE-related genes were discovered near *van* loci in [*Clostridium*] *methoxybenzovorans* SR3 and [*Clostridium*] *indolis* DSM 755.

Clade (V) included a large portion of VanHA sequences from different *Bacilli* spp.; the repertoire of MGE-related genes co-localized with *van* loci was rather poor in species belonging to this clade (Figure 5). Finally, a subclade of clade (V)—(V’)—appeared to be composed of VanHA sequences from plasmids carrying Tn*1546*-like transposons, isolated from clinically relevant enterococci and staphylococci from all over the world (Figure 5, see also ESM Table S1 for references). *Bacillus circulans* VR0709 was a basal taxon of (V’) (Figure 5), where a single MGE-related gene was found adjacent to the *van* loci [50,79]. This was a gene for an IS200/IS605-family (Y1) transposase (CAB61226) that had no homologs in other clade (V’) strains. Peculiarly, *Ecc. faecium* RBWH1 pRBWH1.3^+^ [32] appeared to carry two sets of *van* genes simultaneously: one as a part of the Tn*1546*-like transposon on the plasmid (pRBWH1.3), and another as a part of the Tn*1549*-like transposon integrated into the chromosome.

Concluding the analysis of the tree, it seems interesting to note that VanHA sequences encoded in putative MGEs from two actinobacteria—*Parvibacter* (*Prv.*) *caecicola* DSM 22242 and *Enterorhabdus* (*Enr*.) *mucosicola* NM66_B29—formed the basal branch of the whole tree (Figure 5).

## 4. Discussion

**Figure 6:**
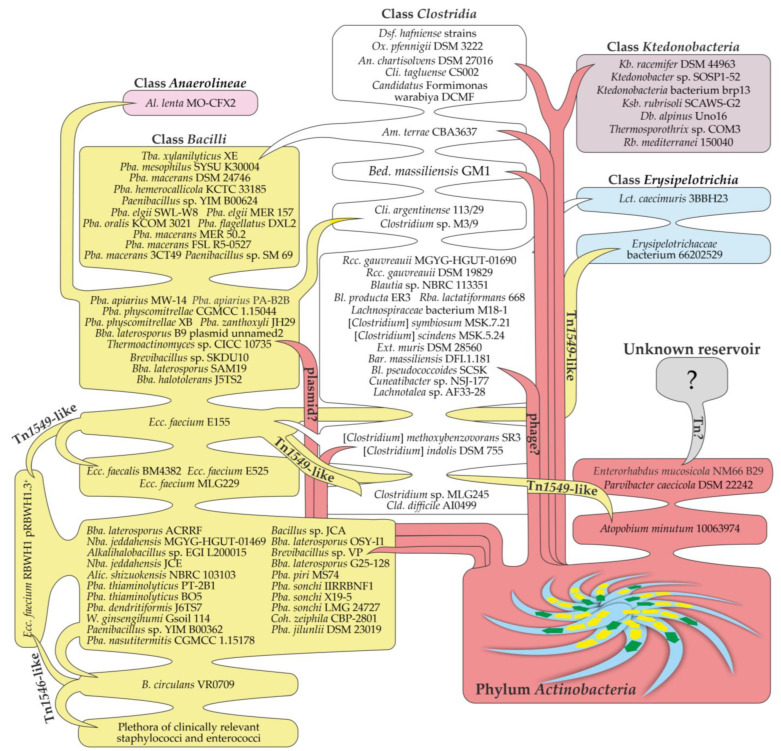
A scheme depicting a possible scenario for the transmission of *vanHAX* genes from *Actinobacteria* spp. to representatives of other classes. The scheme is discussed in the main text.

The results obtained in the current work indicate that *van* genes are more widely distributed among bacteria than expected before: for the first time, *van* genes were discovered in the genomic records of organisms from still poorly studied classes such as *Anaerolineae*, *Erysipelotrichia*, and *Ktedonobacteria*. Most likely, the spread of *van* genes among these different bacteria was determined by multiple HGT events. This idea is not new [26,52,80,81], but the abundance of genomic data available now allows us to outline some of these HGT events more precisely. Although the overall picture is quite puzzling and we are only at the beginning of such types of investigations, in the discussion, we propose a speculative scenario of how *van* genes might have disseminated (Figure 6).

As noted in the previous section, the most recent HGT event may have led to the transmission of *van* genes from actinobacteria to the *Ktedonobacteria* class and to some members of the *Clostridia* class (see scheme in Figure 6). The corresponding proteins did not diverge much from the actinobacterial ones and were found deep in the “core *Actinobacteria*” clade (Figure 5). However, the G-C content of these *van* loci (<50%) is definitely lower than that of actinobacteria (>60%), indicating that the corresponding genes were eventually adapted to the translation machinery of the novel hosts. Most of these *Ktedonobacteria* and *Clostridia* spp. were isolated from soil biotopes [55], likely sharing their ecological niches with *Actinobacteria* spp.—the source of *van* loci. Notable exceptions were *Ox. pfennigii* DSM 3222, isolated from cattle rumen [82] (a biotope also inhabited by different actinobacteria [83]), and *Cli. tagluense* CS002—a causative agent of meat spoilage [84]. Although no MGE-related genes were discovered near *van* loci in *Ox. pfennigii* DSM 3222 and only a single ISNCY-family transposase was encoded near *van* loci in *Cli. tagluense* CS002, these examples chart new possible routes for the further spread of *van* genes in antropocenoses. The vehicle/vehicles of such putative HGT events remain elusive, although multiple genes for transposases and resolvases/recombinases/integrases were found to co-localize with *van* loci in these *Ktedonobacteria* and *Clostridia* spp. It seems that in all of these cases, *van* loci were located on the chromosome; consequently, it is also possible that MGE-related genes might have been partially or completely lost upon integration, eroding the genomic landscapes of putative vehicles carrying *van* genes.

IS-like elements might have played some role in the HGT of *van* loci into *Ktedonobacteria* spp., since, in these microbes, an immense number of transposase genes and intact IS elements are found nearby. Notably, *van* loci in *Ktedonobacteria* spp. underwent peculiar modifications: genes coding for VanX were lost, while novel genes for other types of peptidases were gained. Some evidence exists that at least some of these new genes were introduced as “passenger genes” in IS-like elements (see Section 3.3). As already mentioned above, *Ktedonobacteria* spp. have an actinomycete-like lifestyle [55]. Accordingly, these microbes might have recruited *van* loci for protection against dalbaheptide producers (inhabiting the same biotopes), self-immunity against certain endogenous secondary metabolites, or morphogenesis-related cell-wall remodeling. Overall, the case of *van* genes in *Ktedonobacteria* spp. is of utmost interest and requires further experimental investigation.

Aside from VanHA sequences rooted deep in “Core *Actinobacteria*”, our reference tree demonstrated the presence of five well-defined clades (99% bootstrap support) and a monotaxon branch (*Bed. massiliensis* GM1). For further interpretation, we would like to make two assumptions: (a) each of these clades (and *Bed. massiliensis* GM1 branch) emerged as a result of a single HGT event from *Actinobacteria*; (b) the basal taxa of each clade represent a starting point in which *van* loci were delivered by an initial HGT event (before further spreading to the other taxa of the clade). Following these assumptions, an interesting speculative yet plausible scenario for *van* gene dissemination can be proposed (Figure 6).

In clade (I), a HGT event likely introduced *van* loci to some *Clostridia* spp. since the basal taxon of this clade is *Am. terrae* CBA3637, belonging to the class *Clostridia. Am. terrae* CBA3637 was isolated from river sediments [85], which are also inhabited by a plethora of actinobacteria [86]. Further, *van* genes spread to other clade (I) taxa—*Tba. xylanilyticus* XE and *Paenibacillus* spp. (Figure 6).

Clade (II) points to several HGT events in addition to the putative initial one. Here, the majority of taxa belong to the *Bacilli* class. However, sequences from an *Anaerolineae* class bacterium—*Al. lenta* MO-CFX2—are positioned far from the base of clade (II), suggesting a putative HGT event delivering *van* genes from clade (II) *Bacilli* spp. to *Al. lenta* MO-CFX2. Notably, *Al. lenta* MO-CFX2 is the first reported Gram-negative bacterium [87] carrying *van* loci. The same scenario also works for the two *Clostridia* spp. found in clade (II)—*Clostridium* sp. M3/9 and *Cli. argentinense* 113/29. In at least one clade (II) taxon, *Bba. laterosporus* B9, *van* loci are located on a plasmid, implying that a possible vehicle bringing *van* genes to clade (II) might have been a plasmid.

Except for one, all taxa forming clade (III) belong to *Clostridia* spp. An exception is the VanHA sequences of *Lct. caecimuris* 3BBH23 from the *Erysipelotrichia* class, rooted deep in clade (III). Such a topology most likely indicates that *Lct. caecimuris* 3BBH23 received *van* loci from some clade (III) *Clostridia* spp. as a result of an additional HGT event (Figure 6). It is also important to note that *van* loci in the basal taxon of the clade (III)—*Lachnotalea* sp. AF33-28—are located downstream of an incomplete prophage region. Thus, the vehicles that delivered *van* genes to clade (III) organisms might have been some phages.

VanHA sequences from the classes *Bacilli*, *Erysipelotrichia*, *Clostridia* and the phylum *Actinobacteria* composed clade (IV). The basal branch of clade (IV) was represented by sequences from [*Clostridium*] *indolis* DSM 755 and [*Clostridium*] *methoxybenzovorans* SR3, isolated from soil [88] and wastewater [89], respectively. No MGE-related genes accompanied *van* loci in these two organisms. However, in all of the other taxa of clade (III), *van* genes were found as a part of Tn*1549*-like transposons. Such evidence induces us to speculate that an unknown vehicle delivered *van* genes to [*Clostridium*] *indolis* DSM 755 and [*Clostridium*] *methoxybenzovorans* SR3 (or ancestral organisms) in an initial HGT event, leading to the emergence of clade (III) (Figure 6). In the following HGT events, *van* loci were mobilized by a Tn*1549*-like transposon and then spread to other clade (III) taxa. These taxa included several clinical and human isolates: an organism from the *Erysipelotrichia* class (*Erysipelotrichaceae* bacterium 66202529), *Cld. difficile* AI0499, *Clostridium sp.* MLG245, several strains of enterococci, and the actinobacterium *Atpb. minutum* 10063974 (Figure 6). Overall, we might conclude that two bacteria from the *Erysipelotrichia* class obtained *van* genes in two independent HGT events, while the Tn*1549*-like transposon mediated the reverse transition of *van* genes to the *Actinobacteria* phylum in the case of *Atpb. minutum* 10063974.

Unlike all other clades, clade (V) was composed exclusively of *Bacilli* spp. The basal taxa of clade (V) were mostly soil dwellers and did not show any clear repetitive patterns of MGE-related genes co-localized with *van* loci (Figure 5). Herein, subclade (V’) was the most interesting, being composed principally of VanHA sequences encoded on plasmids carrying *Tn1546*-like transposons from different clinical isolates of staphylococci and enterococci. At the basal branch of subclade (V’), there are VanHA sequences from a clinical isolate of the soil-dwelling opportunistic human pathogen *B. circulans* VR0709 [50]. Overall, the topology of clade (V) suggests that an unknown vehicle delivered *van* loci to *B. circulans* VR0709 from a gene pool existing in basal clade (V) taxa. Consequentially, a Tn*1546*-containing plasmid mobilized *van* loci from *B. circulans* VR0709 and mediated further dissemination in nosocomial environments among staphylococci and enterococci (Figure 6). This last observation actually corroborates a previous conclusion made from a phylogenetic analysis conducted more than ten years ago on a less extensive sample of available *van* gene sequences [80].

Finally, we would also like to comment on *van* genes from the two actinobacterial strains—*Prv. caecicola* DSM 22242 and *Enr. mucosicola* NM66_B29. VanHA sequences from these two organisms were the most divergent, being an outgroup for our reference tree (Figure 5). In our previous work, we demonstrated that *van* loci in *Prv. caecicola* DSM 22242 and *Enr. mucosicola* NM66_B29 are co-localized with an assemblage of MGE-related genes resembling a transposon of unknown type [25]. Notably, both strains were isolated from the intestines of laboratory mice [90,91]. Keeping in mind the case of *Atpb. minutum* 10063974 (see above), it is tempting to suppose that *Prv. caecicola* DSM 22242 and *Enr. mucosicola* NM66_B29 represent another example of a reverse HGT event that brought *van* genes back to the *Actinobacteria* phylum. However, the source of the putative transposons found in *Prv. caecicola* DSM 22242 and *Enr. mucosicola* NM66_B29 remains unknown (Figure 6).

Our results demonstrate that *van* genes from the *Actinobacteria* phylum represent an interesting and widespread part of the *Terrabacteria* mobilome. The flow of *van* genes is a continuous process that occurs actively, as demonstrated by the example of *Ktedonobacteria* spp. Likely carried by different vehicles, *van* genes spread from one taxon to another, creating a puzzling network of consequential HGT events (Figure 6). Bacteria from the *Bacilli* and *Clostridia* classes play an important role in the spread of *van* genes and might represent their source for classes such as *Anaerolineae* and *Erysipelotrichia*. We are aware that our scenario depicting the spread of *vanHAX* genes is speculative and that it will probably change when more genomic information becomes available. However, we believe that at the current point, it provides a useful interpretation of the overall picture to start from in future investigations.

## 5. Conclusions

Through extensive bioinformatic work, we discovered that *van* genes are present in three classes of eubacteria—*Anaerolineae*, *Erysipelotrichia*, and *Ktedonobacteria*—as well as in new genera belonging to the *Clostridia* and *Bacilli* classes. The majority of these *van* genes tended to be co-localized with MGE-related genes of various types. Based on phylogenetic reconstruction, we could assume that the class *Anaerolineae* recently obtained *van* genes from *Bacilli* spp., while two recent independent HGT events delivered *van* genes to *Erysipelotrichia* from *Clostridia* and *Bacilli* spp., respectively. In addition, the most recent HGT event spread *van* genes from *Actinobacteria* directly to species belonging to the *Ktedonobacteria* class. *Ktedonobacteria* tended to lose *vanX* and gain novel genes coding for carboxypeptidases. Finally, a Tn*1549*-like transposon likely mobilized *van* genes from *Clostridia* spp., while a Tn*1546*-like transposon mobilized *van* genes from *Bacilli* spp.

## Figures and Tables

**Table 1:** List of bacterial strains from *Anaerolineae*, *Bacilli*, *Clostridia*, *Erysipelotrichia*, and *Ktedonobacteria* classes found to carry *van* loci; more detailed information, as well as accession numbers for nucleic and aa sequences, is given in Supplementary Excel File S1.

Classes:	Genera:	Bacterial Strains Carrying *van* Genes:
*Anaerolineae*	*Aggregatilinea* (*Al*.)	*Aggregatilinea lenta* MO-CFX2
*Bacilli*	*Alicyclobacillus* (*Alic*.)	*Alicyclobacillus shizuokensis* NBRC 103103
	*Alkalihalobacillus*	*Alkalihalobacillus* sp. EGI L200015
	*Anoxybacillus* (*Anox.*)	*Anoxybacillus sediminis* PCH 117
	*Bacillus*	*Bacillus* sp. JCA
	*Brevibacillus* (*Bba.*)	*Brevibacillus halotolerans* J5TS2; *Brevibacillus laterosporus* ACRRF; *Bba. laterosporus* G25-128; *Bba. laterosporus* OSY-I1; *Bba. laterosporus* SAM19; *Bba. laterosporus* B9 (plasmid); *Brevibacillus* sp. MCWH; *Brevibacillus* sp. NL20B1; *Brevibacillus* sp. SKDU10; *Brevibacillus* sp. VP
	*Cohnella* (*Coh.*)	*Cohnella zeiphila* CBP-2801
	*Neobacillus* (*Nba.*)	*Neobacillus jeddahensis* MGYG-HGUT-01469; *Nba. jeddahensis* JCE
	*Paenibacillus* (*Pba.*)	*Paenibacillus apiarius* MW-14; *Paenibacillus dendritiformis* J6TS7; *Paenibacillus elgii* MER 157; *Pba. elgii* SWL-W8; *Paenibacillus* sp. DXL2; *Paenibacillus hemerocallicola* KCTC 33185; *Paenibacillus jilunlii* DSM 23019; *Paenibacillus macerans* 3CT49; *Pba. macerans* DSM 24746; *Pba. macerans* HMSSN-036; *Pba. macerans* FSL R5-0527; *Pba. macerans* MER 50.2; *Paenibacillus mesophilus* SYSU K30004; *Paenibacillus nasutitermitis* CGMCC 1.15178; *Paenibacillus oralis* KCOM 3021; *Paenibacillus physcomitrellae* CGMCC 1.15044; *Pba. physcomitrellae* XB; *Paenibacillus piri* MS74; *Paenibacillus sonchi* IIRRBNF1; *Pba. sonchi* X19-5; *Paenibacillus* sp. cl123; *Paenibacillus* sp. SM 69; *Paenibacillus* sp. UNCCL117; *Paenibacillus* sp. YIM B00362; *Paenibacillus* sp. YIM B00624; *Paenibacillus thiaminolyticus* BO5; *Paenibacillus zanthoxyli* JH29; *Paenibacillus flagellatus* DXL2
	*Thermoactinomyces*	*Thermoactinomyces* sp. CICC 10735
	*Thermobacillus* (*Tba.*)	*Thermobacillus xylanilyticus* XE
	*Weizmannia* (*W.*)	*Weizmannia ginsengihumi* Gsoil 114
*Clostridia*	Dubious genus	[*Clostridium*] *indolis* DSM 755; [*Clostridium*] *methoxybenzovorans* SR3; [*Clostridium*] *scindens* MSK.5.24; [*Clostridium*] *symbiosum* MSK.7.21
	Unidentified genus	*Lachnospiraceae* bacterium M18-1
	*Aminipila* (*Am*.)	*Aminipila terrae* CBA3637
	*Anaerobacterium* (*An.*)	*Anaerobacterium chartisolvens* DSM 27016
	*Bariatricus* (*Bar.*)	*Bariatricus massiliensis* DFI.1.181
	*Beduini* (*Bed*.)	*Beduini massiliensis* GM1
	*Blautia* (*Bl.*)	*Blautia producta* ER3; *Blautia pseudococcoides* SCSK; *Blautia* sp. NBRC 113351
	*Candidatus* Formimonas	*Candidatus* Formimonas warabiya DCMF
	*Clostridium* (*Cli.*)	*Clostridium argentinense* 113/29; *Clostridium* sp. M3/9; *Clostridium tagluense* CS002
	*Cuneatibacter*	*Cuneatibacter* sp. NSJ-177
	*Extibacter* (*Ext.*)	*Extibacter muris* DSM 28560
	*Lachnotalea*	*Lachnotalea* sp. AF33-28
	*Oxobacter* (*Ox.*)	*Oxobacter pfennigii* DSM 3222
	*Ruminococcus* (*Rcc*.)	*Ruminococcus gauvreauii* DSM 19829; *Ruminococcus gauvreauii* MGYG-HGUT-01690
	*Ruthenibacterium* (*Rba.*)	*Ruthenibacterium lactatiformans* 668
*Erysipelotrichia*	Unidentified genus	*Erysipelotrichaceae* bacterium 66202529
	*Longicatena* (*Lct.*)	*Longicatena caecimuris* 3BBH23
*Ktedonobacteria*	Unidentified genus	*Ktedonobacteria* bacterium brp13
	*Dictyobacter* (*Db.*)	*Dictyobacter alpinus* Uno16
	*Ktedonobacter* (*Kb.*)	*Ktedonobacter racemifer* DSM 44963; *Ktedonobacter* sp. SOSP1-52
	*Ktedonosporobacter* (*Ksb.*)	*Ktedonosporobacter rubrisoli* SCAWS-G2
	*Reticulobacter* (*Rb.*)	*Reticulibacter mediterranei* 150040
	*Thermosporothrix*	*Thermosporothrix* sp. COM3

## Data Availability

All data obtained in the study are presented in either the main text or Supplementary Materials.

## Supplementary Materials

The following are available online at https://www.mdpi.com/article/10.3390/genes13111960/s1.
