# Supplementary material for: Occurrence of vanHAX and Related Genes beyond the Actinobacteria Phylum
Source: Genes (Basel). 2022 Oct 27;13(11):1960. doi: 10.3390/genes13111960 (PMC9689679; doi:10.3390/genes13111960)
Supplement: Supplementary file 1 [file genes-13-01960-s001.zip › Yushchuk et al ESM.pdf]

Electronic Supplementary Materials

Occurrence of *vanHAX* and related genes beyond the *Actinobacteria* phylum

Oleksandr Yushchuk [1,2], Elisa Binda [1], Victor Fedorenko [2] and Flavia Marinelli [1,*]

[1] Department of Biotechnology and Life Sciences, University of Insubria, Varese, Italy

[2] Department of Genetics and Biotechnology, Ivan Franko National University of Lviv, Lviv, Ukraine

**Correspondence:* flavia.marinelli@uninsubria.it; Tel.: +39-0332-42-1546

Inventory of Electronic supplementary materials

Contents	Page
**ESM Table S1.** List of the bacterial species carrying *vanHAXRS* genes beyond *Actinobacteria* phylum reported in the literature	**3**
**ESM Figure S1.** Phylogenetic tree of VanH proteins from *Actinobacteria, Anaerolineae, Bacilli, Clostridia, Erysipelotrichia* and *Ktedonobacteria*	**5-7**
**ESM Figure S2.** Phylogenetic tree of VanA proteins from *Actinobacteria, Anaerolineae, Bacilli, Clostridia, Erysipelotrichia* and *Ktedonobacteria*	**8-10**
**ESM Figure S3.** Phylogenetic tree of VanHA sequences from *Actinobacteria, Anaerolineae, Bacilli, Clostridia, Erysipelotrichia* and *Ktedonobacteria*	**11-12**
**Supplementary References**	**13**

ESM Table S1. List of the bacterial species beyond *Actinobacteria* phylum described in the literature and from which sequencing data for *vanHAXRS* genes are available.

**Taxonomic position**	**Accession number of corresponding nucleic acid sequence**	**Strain carrying *vanHAXRS* genes**	**Reference**
Class ***Bacilli***	CP022486	*Enterococcus* (*Ecc.*) *faecalis* ARO1/DG, plasmid pARO1.3	[1]
	PRJNA476469	Multiple *Ecc. faecalis* isolates, short reads covering parts of *van* genes available	[1]
	AB247327	*Ecc. faecalis*, plasmid pSL1	[2]
	GQ484956	*Ecc. faecalis*, plasmid pWZ1668	[3]
	GQ484955	*Ecc. faecalis*, plasmid pWZ7140	[3]
	GQ484954	*Ecc. faecalis*, plasmid pWZ909	[3]
	AF192329	*Ecc. faecalis* BM4382, transposon Tn*1549*	[4]
	NZ_AUWX01000187	*Ecc. faecium* E155	[5]
	NZ_AUWV01000199	*Ecc. faecium* E525	[5]
	FN424376	*Ecc. faecium* 399/F98/A4, partial sequence of plasmid pVEF4	[6]
	AM932524	*Ecc. faecium* BM4147, plasmid pIP816	[6]
	HQ115078	*Ecc. faecium* S177, plasmid pS177	[7]
	JQ663598	*Ecc. faecium*, plasmid pF856	[8]
	AM296544	*Ecc. faecium* 399/F99/H8, plasmid pVEF1	[9]
	AM410096	*Ecc. faecium* 399/F99/A9, plasmid pVEF2	[9]
	AM931300	*Ecc. faecium* 399/S99/A7, plasmid pVEF3	[10]
	NZ_RDPZ01000021, NZ_RDPZ01000080	*Ecc. faecium* HBSJRP	[11]
	AY655721	*Ecc. faecium* MLG229	[12]
	NZ_CP064436	*Ecc. faecium* PR00859-7, plasmid unnamed_4	[13]
	NZ_CP064428	*Ecc. faecium* PR01996-12 plasmid unnamed_4	[13]
	NZ_CP064448	*Ecc. faecium* PR02395-7 plasmid unnamed_5	[13]
	NZ_CP064419	*Ecc. faecium* PR02648-8 plasmid unnamed_2	[13]
	NZ_CP033206	*Ecc. faecium* RBWH1	[14]
	NZ_CP033209	*Ecc. faecium* RBWH1, plasmid pRBWH1.3	[14]
	NZ_JSET01000031	*Ecc. faecium* VRE3	[15]
	KY595962	*Ecc. faecium* VREfm1, plasmid pPEC286	[16]
	KY595966	*Ecc. faecium* VREfm81, plasmid pBUD102	[16]
	KY595968	*Ecc. faecium* VREfm87, plasmid pMIS10	[16]
	CM003134	*Ecc. gallinarum* A6981, plasmid pA6981	[17]
	PRJNA604849	*Enterococcus* sp. isolates, short reads covering parts of *van* genes	[18]
	AE017171	*Staphylococcus* (*Scc.*) *aureus*, plasmid pLW043	[19]
	NZ_LJOB01000059	*Scc. aureus* HOU1444-VR	[20]
	NZ_CP012594	*Scc. aureus* strain HOU1444-VR, plasmid pVR-MSSA_01	[20]
	NZ_AHBK01000092	*Scc. aureus* ssp. *aureus* VRS1	[21]
	NZ_AHBT01000037	*Scc. aureus* ssp. *aureus* VRS10	[21]

	NZ_AHBU01000043	*Scc. aureus* ssp. *aureus* VRS11a	[21]
	NZ_AHBV01000045	*Scc. aureus* ssp. *aureus* VRS11b	[21]
	NZ_AHBL01000065	*Scc. aureus* ssp. *aureus* VRS2	[21]
	NZ_AHBM01000055	*Scc. aureus* ssp. *aureus* VRS3a	[21]
	NZ_AHBN01000064	*Scc. aureus* ssp. *aureus* VRS4	[21]
	NZ_AHBO01000060	*Scc. aureus* ssp. *aureus* VRS5	[21]
	NZ_AHBP01000061	*Scc. aureus* ssp. *aureus* VRS6	[21]
	NZ_AHBQ01000050	*Scc. aureus* ssp. *aureus* VRS7	[21]
	NZ_AHBR01000049	*Scc. aureus* ssp. *aureus* VRS8	[21]
	NZ_AHBS01000053	*Scc. aureus* ssp. *aureus* VRS9	[21]
	DQ018711	*Paenibacillus* (*Pba.*) *apiarius* PA-B2B	[22]
	AF155139	*Pba. popilliae* ATCC 14706	[23]
	DQ018710	*Pba. thiaminolyticus* PT-2B1	[22]
	CP068595	*Pba. sonchi* LMG 24727	[24]
	CP019652	*Pba. larvae* subsp. *larvae* DSM 25430	[24]
	CP020557	*Pba. larvae* subsp. *pulvifaciens* strain SAG 10367	[24]
	CP019687	*Pba. larvae* subsp. *larvae* strain ATCC 9545	[24]
	CP020327	*Pba. larvae* subsp. *pulvifaciens* strain CCM 38	[24]
	CP019794	*Pba. larvae* subsp. *pulvifaciens* strain ATCC 13537	[24]
	CP003355	*Pba. larvae* subsp. *larvae* DSM 25430	[24]
	CP019717	*Pba. larvae* subsp. *larvae* strain Eric_V	[24]
	CP032410	*Brevibacillus* (*Bba.*) *laterosporus* E7593-50	[24]
	CP039710	*Thermoactinomyces* (*Tam.*) *vulgaris* 2H	[24]
	CP036487	*Tam. vulgaris* CDF	[24]
	Y15704-08, Y17303-05	*Bacillus* (*B.*) *circulans* VR0709	[25]
Class ***Clostridia***	KU558763	*Clostridioides* (*Cld.*) *difficile* AI0499, transposon Tn*1549*-like	[26]
	AY655720	*Clostridium* (*Cli.*) sp. MLG245, transposon Tn*1549*-like	[12]
	AP008230<br>JH414450<br>CP001336	*Desulfitobacterium (Dsf.) hafniense* Y51<br>*Dsf. hafniense* DP7<br>*Dsf. hafniense* DCB-2	[27]

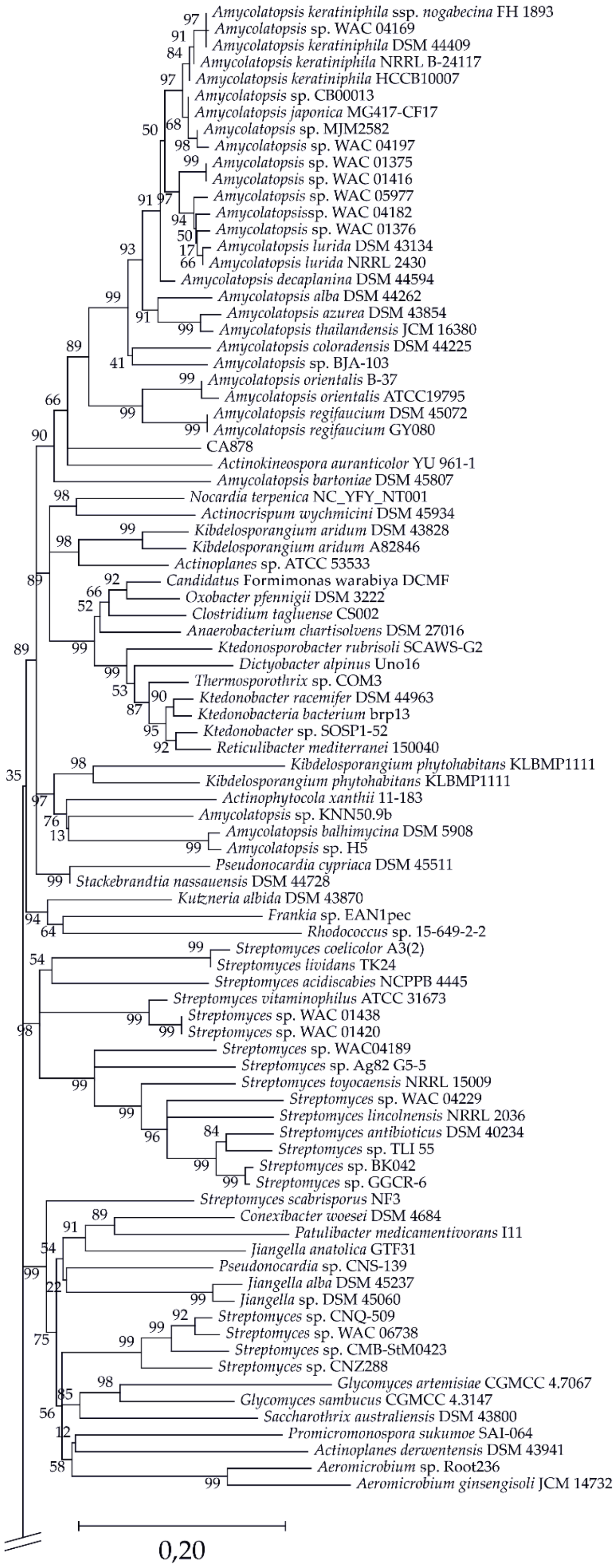

ESM Figure S1. Continued on the next page.

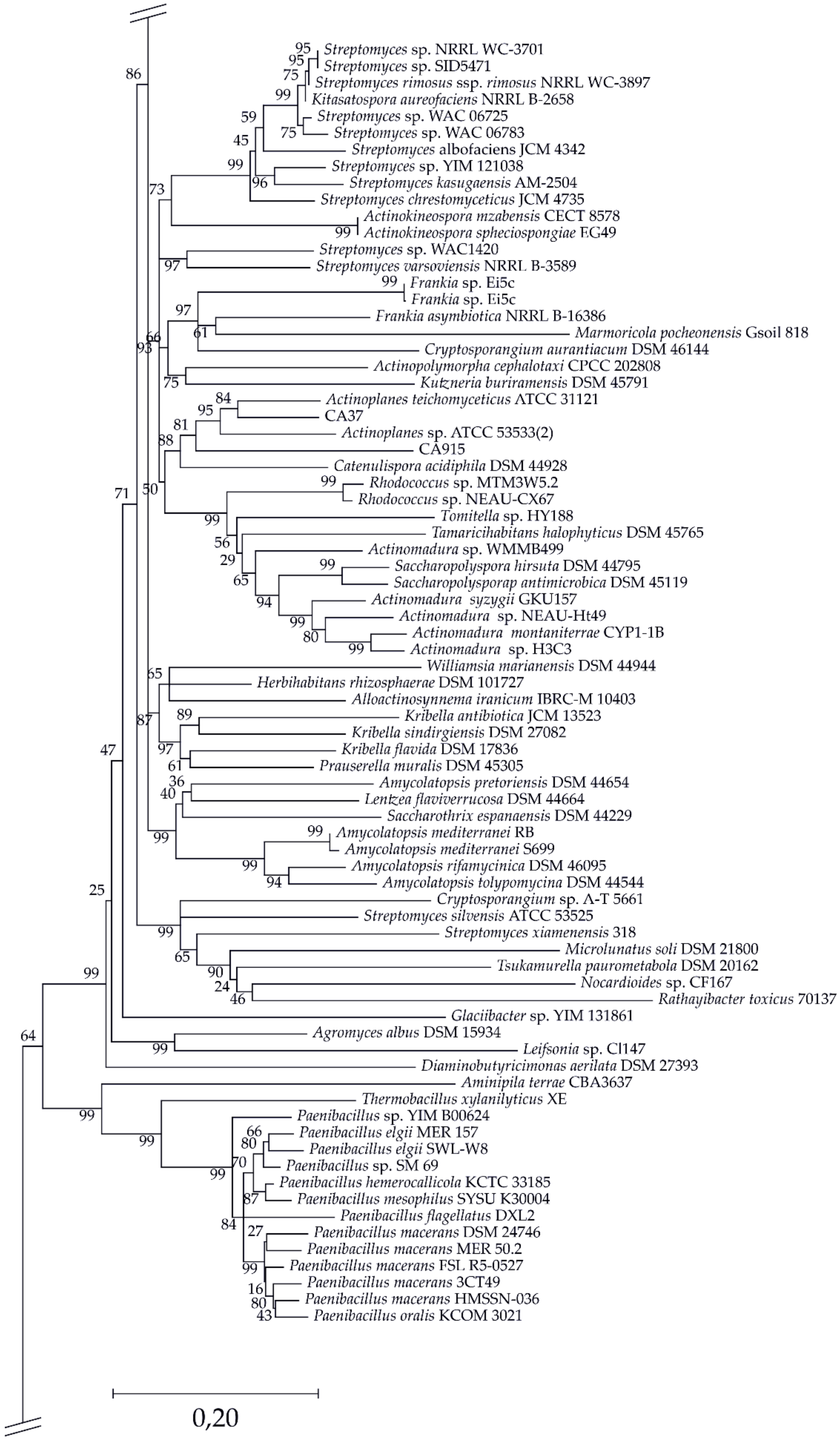

ESM Figure S1. Continued on the next page.

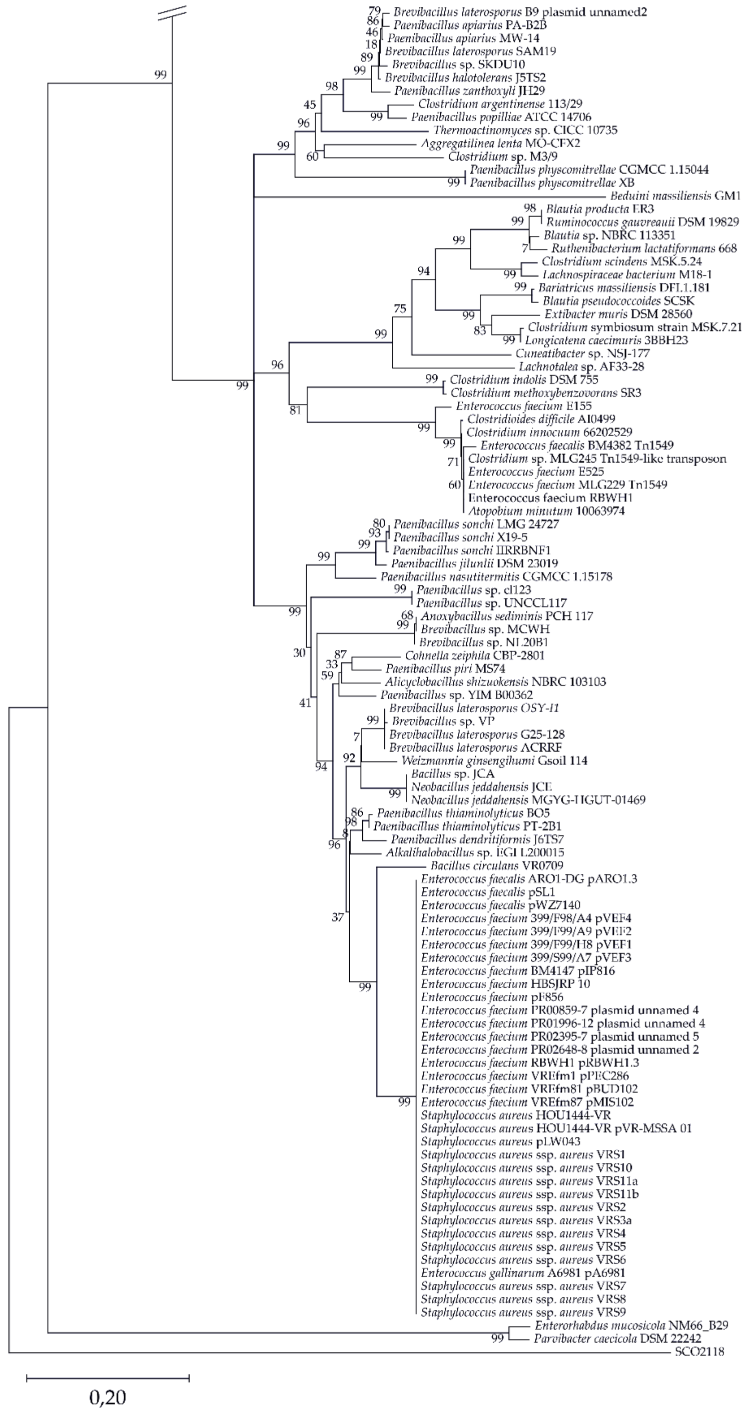

ESM Figure S1. Rooted neigbour-joining phylogenetic tree showing the overall phylogeny of 269 VanH-proteins coming from *Actinobacteria, Anaerolineae, Bacilli, Clostridia, Erysipelotrichia,* and *Ktedonobacteria,* including SCO2118 – a putative D-Lac dehydrogenase – from *Streptomyces coelicolor* A3(2) as an outgroup. Approach for the reconstruction of the tree is described in Methods section. Scale bar represents number of substitutions per site.

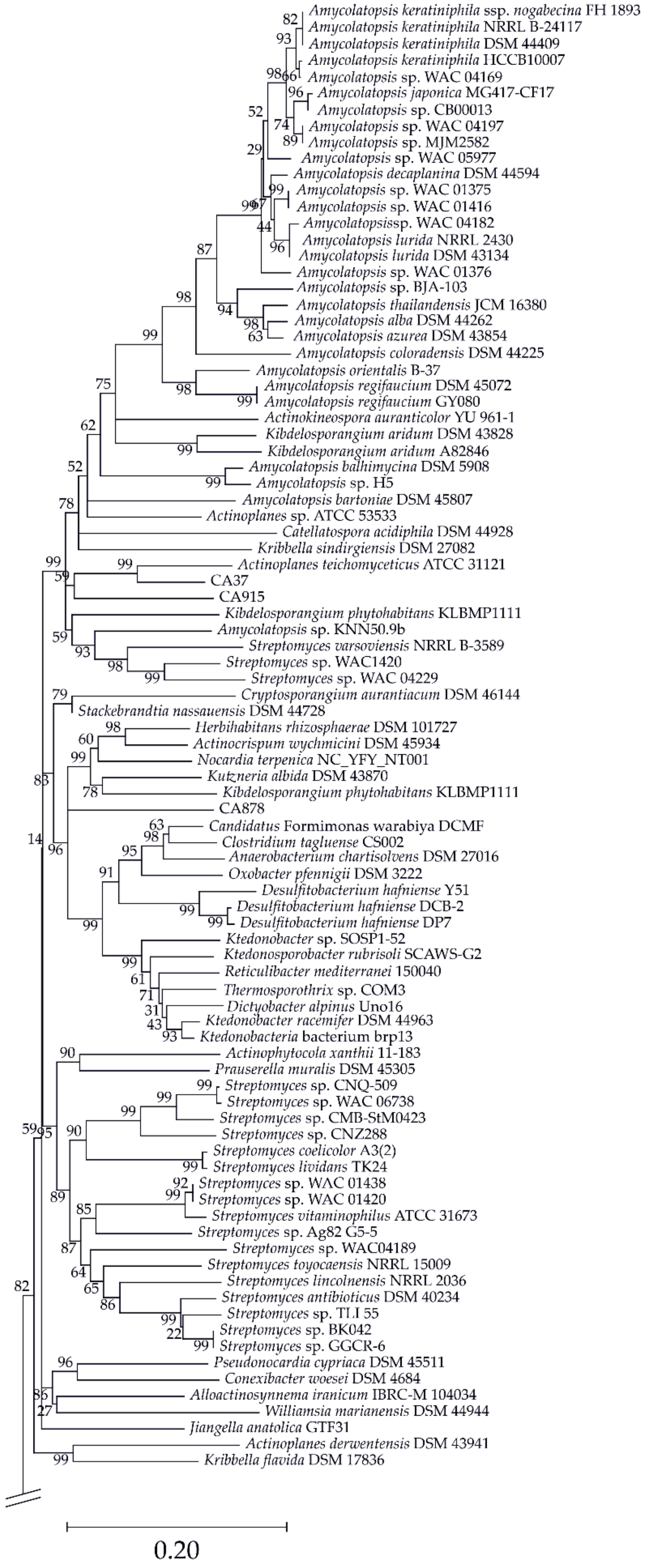

ESM Figure S2. Continued on the next page.

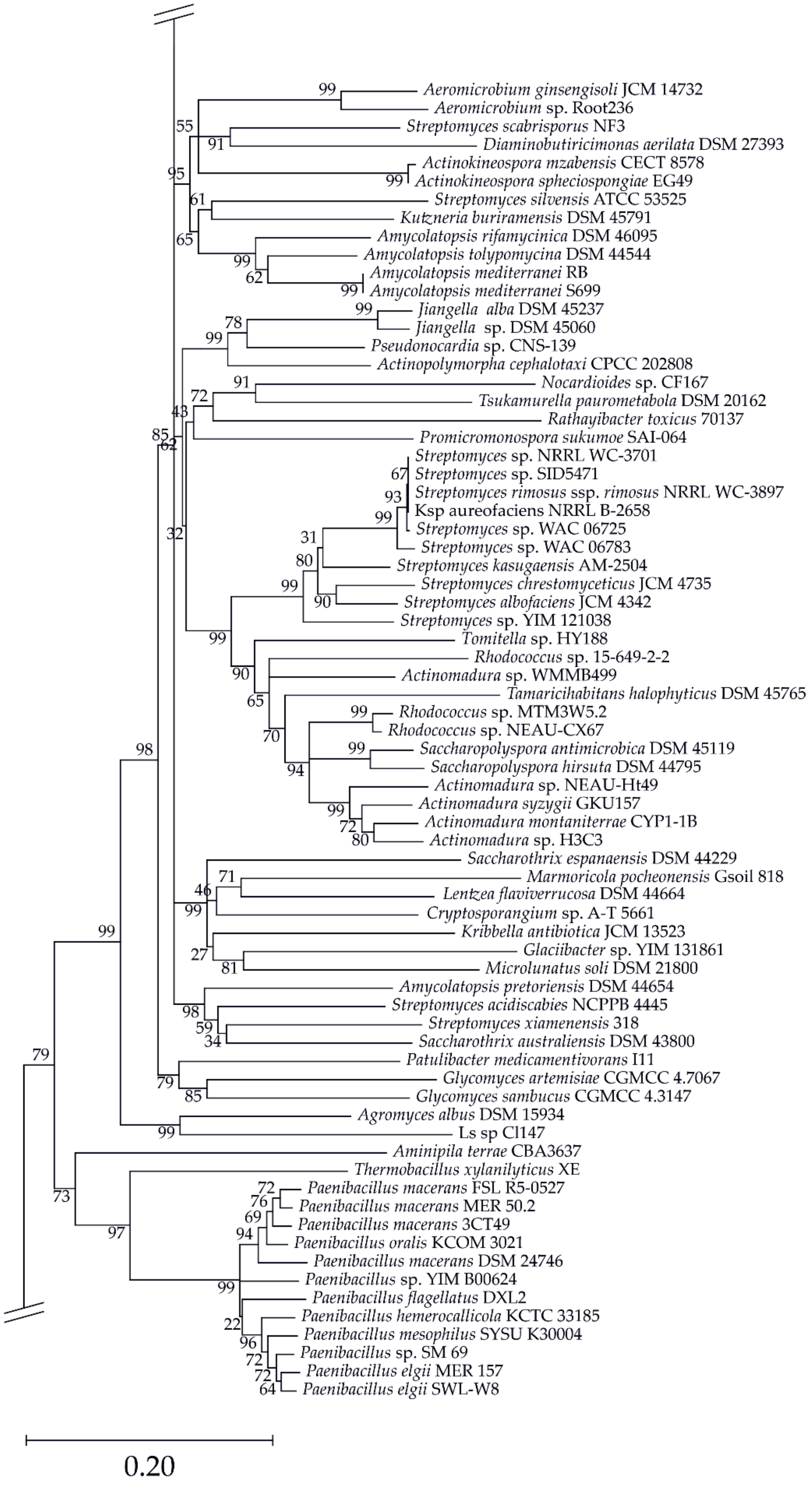

ESM Figure S2. Continued on the next page.

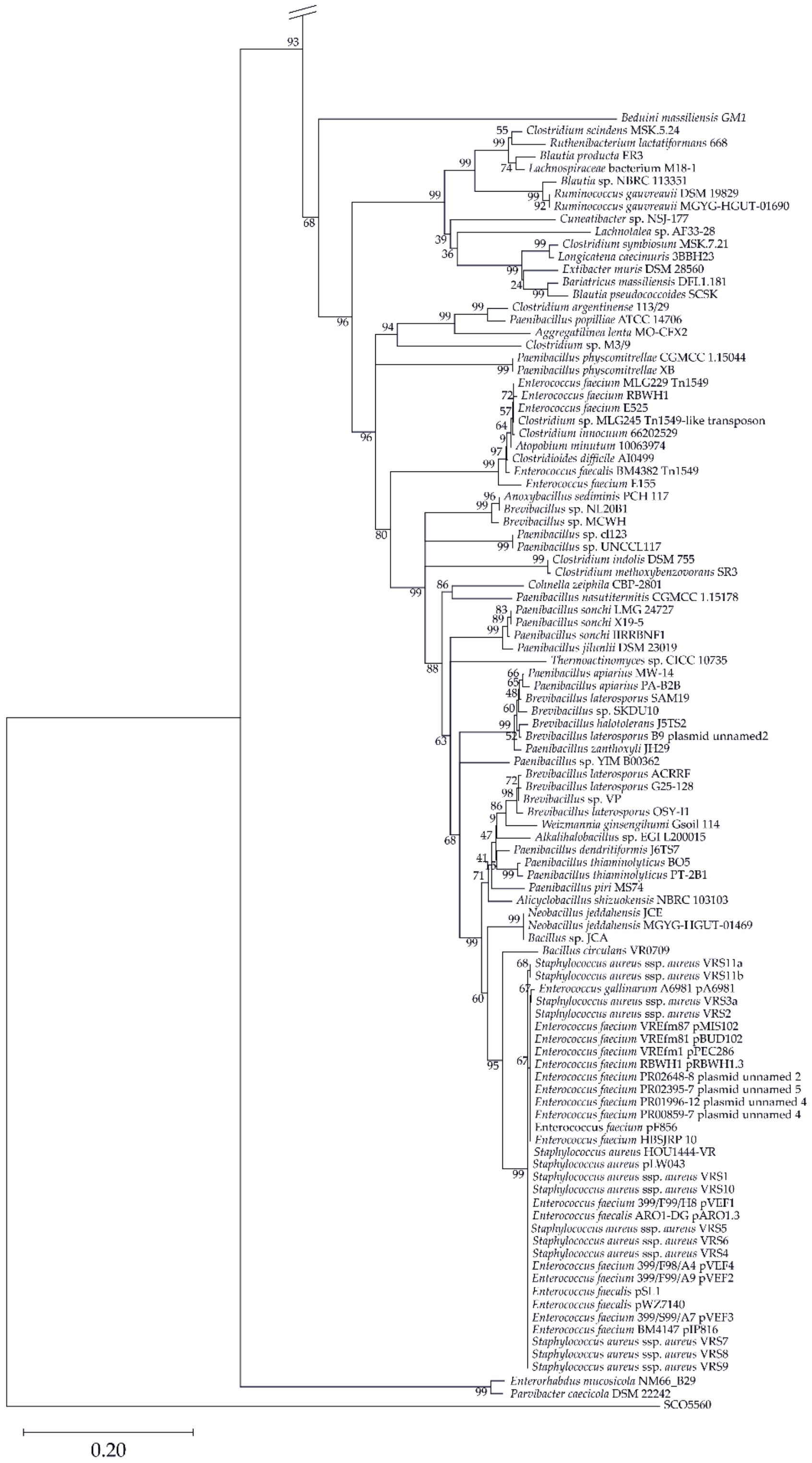

ESM Figure S2. Rooted neigbour-joining phylogenetic tree showing the overall phylogeny of 265 VanA-proteins coming from *Actinobacteria, Anaerolineae, Bacilli, Clostridia, Erysipelotrichia,* and *Ktedonobacteria,* including SCO5560 – a putative D-Ala-D-Ala ligase – from *S. coelicolor* A3(2) as an outgroup. Approach for the reconstruction of the tree is described in Methods section. Scale bar represents number of substitutions per site.

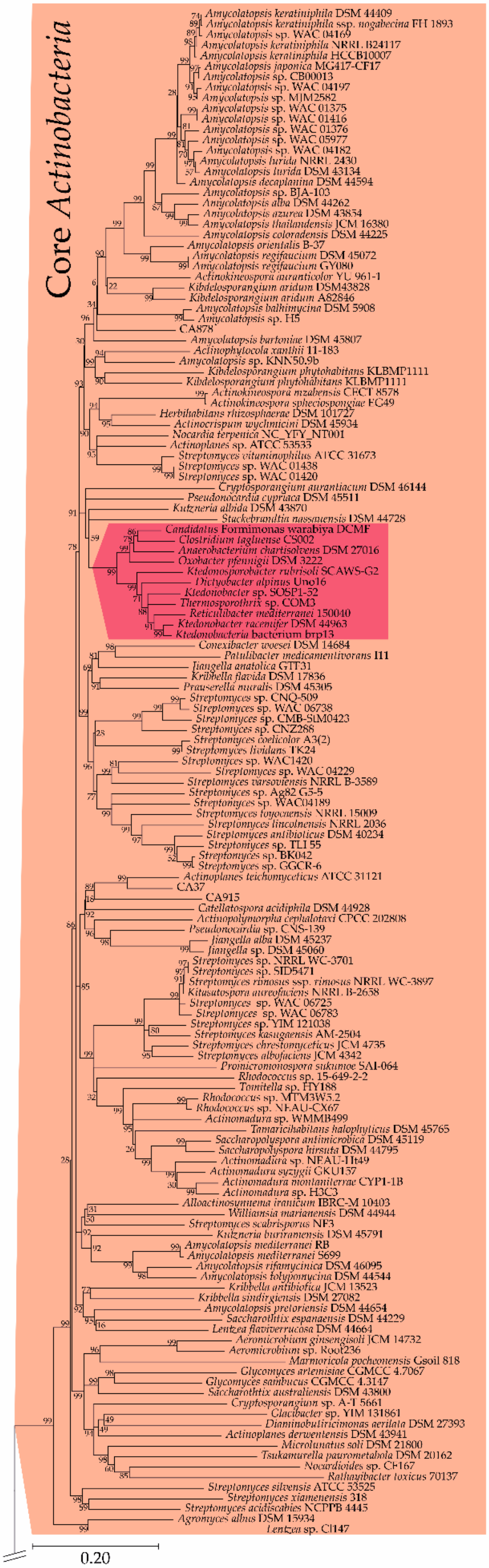

ESM Figure S3. Continued on the next page.

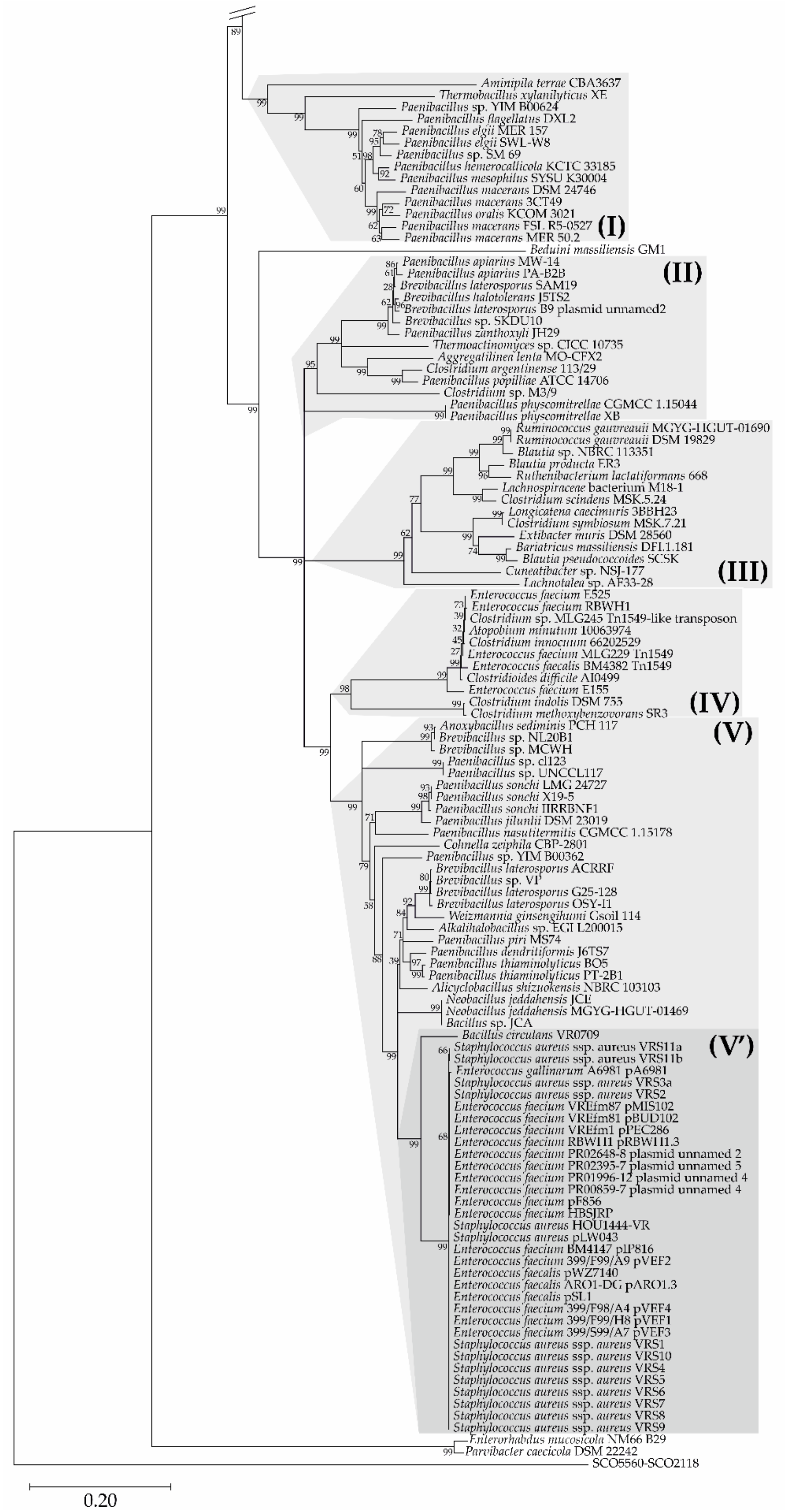

ESM Figure S3. A version of a main text Figure 4 drawn to scale. Scale bar represents number of substitutions per site. "Core *Actinobacteria*" clade is highlighted in pink, while *Clostridia* and *Ktedonobacteria* subclade is in dark pink. Clades (I-V) discussed in main text are highlighted in grey.

Supplementary References

1. Rushton-Green, R.; Darnell, R.L.; Taiaroa, G.; Carter, G.P.; Cook, G.M.; Morgan, X.C. Agricultural origins of a highly persistent lineage of vancomycin-resistant *Enterococcus faecalis* in New Zealand. *Appl. Environ. Microbiol.* **2019**, *85*, e00137-19, 1-17, doi:10.1128/AEM.00137-19.

2. Lim, S.K.; Tanimoto, K.; Tomita, H.; Ike, Y. Pheromone-responsive conjugative vancomycin resistance plasmids in *Enterococcus faecalis* isolates from humans and chicken feces. *Appl. Environ. Microbiol.* **2006**, *72*, 6544–6553, doi:10.1128/AEM.00749-06.

3. Zhu, W.; Murray, P.R.; Huskins, W.C.; Jernigan, J.A.; McDonald, L.C.; Clark, N.C.; Anderson, K.F.; McDougal, L.K.; Hageman, J.C.; Olsen-Rasmussen, M.; et al. Dissemination of an *Enterococcus Inc18*-like *vanA* plasmid associated with vancomycin-resistant *Staphylococcus aureus*. *Antimicrob. Agents Chemother.* **2010**, *54*, 4314–4320, doi:10.1128/AAC.00185-10.

4. Garnier, F.; Taourit, S.; Glaser, P.; Courvalin, P.; Galimand, M. Characterization of transposon Tn*1549*, conferring VanB-type resistance in *Enterococcus* spp. *Microbiology*. **2000**, *146*, 1481–1489, doi:10.1099/00221287-146-6-1481.

5. De Been, M.; Van Schaik, W.; Cheng, L.; Corander, J.; Willems, R.J. Recent recombination events in the core genome are associated with adaptive evolution in *Enterococcus faecium*. *Genome Biol. Evol.* **2013**, *5*, 1524–1535, doi:10.1093/gbe/evt111.

6. Sletvold, H.; Johnsen, P.J.; Wikmark, O.G.; Simonsen, G.S.; Sundsfjord, A.; Nielsen, K.M. Tn*1546* is part of a larger plasmid-encoded genetic unit horizontally disseminated among clonal *Enterococcus faecium* lineages. *J. Antimicrob. Chemother.* **2010**, *65*, 1894–1906, doi:10.1093/jac/dkq219.

7. Halvorsen, E.M.; Williams, J.J.; Bhimani, A.J.; Billings, E.A.; Hergenrother, P.J. Txe, an endoribonuclease of the enterococcal Axe-Txe toxin-antitoxin system, cleaves mRNA and inhibits protein synthesis. *Microbiology* **2011**, *157*, 387–397, doi:10.1099/mic.0.045492-0.

8. Szakacs, T.A.; Kalan, L.; McConnell, M.J.; Eshaghi, A.; Shahinas, D.; McGeer, A.; Wright, G.D.; Low, D.E.; Patel, S.N. Outbreak of vancomycin-susceptible *Enterococcus faecium* containing the wild-type *vanA* gene. *J. Clin. Microbiol.* **2014**, *52*, 1682–1686, doi:10.1128/JCM.03563-13.

9. Sletvold, H.; Johnsen, P.J.; Simonsen, G.S.; Aasnæs, B.; Sundsfjord, A.; Nielsen, K.M. Comparative DNA analysis of two *vanA* plasmids from *Enterococcus faecium* strains isolated from poultry and a poultry farmer in Norway. *Antimicrob. Agents Chemother.* **2007**, *51*, 736–739, doi:10.1128/AAC.00557-06.

10. Sletvold, H.; Johnsen, P.J.; Hamre, I.; Simonsen, G.S.; Sundsfjord, A.; Nielsen, K.M. Complete sequence of Enterococcus faecium pVEF3 and the detection of an ω-ε-ζ toxin-antitoxin module and an ABC transporter. Plasmid. 2008, 60, 75–85, doi:10.1016/j.plasmid.2008.04.002.

11. Mello, S.S.; Van Tyne, D.; Lebreton, F.; Silva, S.Q.; Nogueira, M.C.L.; Gilmore, M.S.; Camargo, I.L.B.C. A mutation in the glycosyltransferase gene *lafB* causes daptomycin hypersusceptibility in *Enterococcus faecium*. *J. Antimicrob. Chemother.* **2020**, *75*, 36–45, doi:10.1093/jac/dkz403.

12. Ballard, S.A.; Pertile, K.K.; Lim, M.; Johnson, P.D.R.; Grayson, M.L. Molecular characterization of *vanB* elements in naturally occurring gut anaerobes. *Antimicrob. Agents Chemother.* **2005**, *49*, 1688–1694, doi:10.1128/AAC.49.5.1688-1694.2005.

13. Kinnear, C.L.; Hansen, E.; Morley, V.J.; Tracy, K.C.; Forstchen, M.; Read, A.F.; Woods, R.J. Daptomycin treatment impacts resistance in off-target populations of vancomycin-resistant *Enterococcus faecium*. *PLoS Biol.* **2020**, *18*, 1–21, doi:10.1371/journal.pbio.3000987.

14. Bohlmann, L.; De Oliveira, D.M.P.; El-Deeb, I.M.; Brazel, E.B.; Harbison-Price, N.; Ong, C.L.Y.; Rivera-Hernandez, T.; Ferguson, S.A.; Cork, A.J.; Phan, M.D.; et al. Chemical synergy between ionophore PBT2 and zinc reverses antibiotic resistance. *MBio* **2018**, *9*, e02391-18, 1-12, doi:10.1128/mBio.02391-18.

15. Khan, S.; Sung, K.; Marasa, B.; Min, S.; Kweon, O.; Nawaz, M.; Cerniglia, C. Draft genome sequence of multidrug-resistant *Enterococcus faecium* clinical isolate VRE3, with a sequence type 16 pattern and novel structural arrangement of Tn*1546*. *Genome Announc.* **2015**, *3*, 1000000, doi:10.1128/genomeA.00871-15.

16. Melegh, S.; Nyul, A.; Kovács, K.; Kovács, T.; Ghidán, Á.; Dombrádi, Z.; Szabó, J.; Berta, B.; Lesinszki, V.; Pászti, J.; et al. Dissemination of VanA-type *Enterococcus faecium* isolates in Hungary. *Microb. Drug Resist.* **2018**, *24*, 1376–1390, doi:10.1089/mdr.2017.0296.

17. Eshaghi, A.; Shahinas, D.; Li, A.; Kariyawasam, R.; Banh, P.; Desjardins, M.; Melano, R.G.; Patel, S.N. Characterization of an *Enterococcus gallinarum* isolate carrying a dual *vanA* and *vanB* cassette. *J. Clin. Microbiol.* **2015**, *53*, 2225–2229, doi:10.1128/JCM.03267-14.

18. Zaheer, R.; Cook, S.R.; Barbieri, R.; Goji, N.; Cameron, A.; Petkau, A.; Polo, R.O.; Tymensen, L.; Stamm, C.; Song, J.; et al. Surveillance of *Enterococcus* spp. reveals distinct species and antimicrobial resistance diversity across a One-Health continuum. *Sci. Rep.* **2020**, *10*, 1–16, doi:10.1038/s41598-020-61002-5.

19. Weigel, L.M.; Clewell, D.B.; Gill, S.R.; Clark, N.C.; McDougal, L.K.; Flannagan, S.E.;

Kolonay, J.F.; Shetty, J.; Killgore, G.E.; Tenover, F.C. Genetic analysis of a high-level vancomycin-resistant isolate of *Staphylococcus aureus*. *Science.* **2003**, *302*, 1569–1571, doi:10.1126/science.1090956.

20. Panesso, D.; Planet, P.J.; Diaz, L.; Hugonnet, J.E.; Tran, T.T.; Narechania, A.; Munita, J.M.; Rincon, S.; Carvajal, L.P.; Reyes, J.; et al. Methicillin-susceptible, vancomycin-resistant *Staphylococcus aureus*, Brazil. *Emerg. Infect. Dis.* **2015**, *21*, 1844–1848, doi:10.3201/eid2110.141914.

21. Kos, V.N.; Desjardins, C. a; Griggs, A.; Cerqueira, G.; Tonder, A. Van; Holden, M.T.G.; Godfrey, P.; Palmer, K.L.; Bodi, K.; Mongodin, E.F.; et al. Comparative genomics of vancomycin-resistant *Staphylococcus aureus* strains and their positions within the clade most commonly associated with methicillin-resistant *S. aureus* hospital-acquired infection in the United States. *MBio.* **2012**, *3*, e00112-12, 1–9, doi:10.1128/mBio.00112-12.Editor.

22. Guardabassi, L.; Perichon, B.; Van Heijenoort, J.; Blanot, D.; Courvalin, P. Glycopeptide resistance *vanA* operons in *Paenibacillus* strains isolated from soil. *Antimicrob. Agents Chemother.* **2005**, *49*, 4227–4233, doi:10.1128/AAC.49.10.4227-4233.2005.

23. Fraimow, H.; Knob, C.; Herrero, I.A.; Patel, R. Putative VanRS-like two-component regulatory system associated with the inducible glycopeptide resistance cluster of *Paenibacillus popilliae*. *Antimicrob. Agents Chemother.* **2005**, *49*, 2625–2633, doi:10.1128/AAC.49.7.2625-2633.2005.

24. Andreo-Vidal, A.; Binda, E.; Fedorenko, V.; Marinelli, F.; Yushchuk, O. Genomic insights into the distribution and phylogeny of glycopeptide resistance determinants within the actinobacteria phylum. *Antibiotics* **2021**, *10*, 1533, 1-30, doi:10.3390/antibiotics10121533.

25. Ligozzi, M.; Lo Cascio, G.; Fontana, R. *vanA* gene cluster in a vancomycin-resistant clinical isolate of *Bacillus circulans*. *Antimicrob. Agents Chemother*. **1998**, *42*, 2055–2059, doi:10.1128/aac.42.8.2055.

26. Knight, D.R.; Androga, G.O.; Ballard, S.A.; Howden, B.P.; Riley, T. V. A phenotypically silent *vanB2* operon carried on a *Tn1549*-like element in *Clostridium difficile*. *mSphere*. **2016**, *1*, e00177-16, 1-4 doi:10.1128/msphere.00177-16.

27. Kruse, T.; Levisson, M.; de Vos, W.M.; Smidt, H. *vanI*: a novel D-Ala-D-Lac vancomycin resistance gene cluster found in *Desulfitobacterium hafniense*. *Microb. Biotechnol.* **2014**, *7*, 456–466, doi:10.1111/1751-7915.12139.
